# Mechanistic insights from structure of *Mycobacterium smegmatis* topoisomerase I with ssDNA bound to both N- and C-terminal domains

**DOI:** 10.1093/nar/gkaa201

**Published:** 2020-03-30

**Authors:** Nan Cao, Kemin Tan, Xiaobing Zuo, Thirunavukkarasu Annamalai, Yuk-Ching Tse-Dinh

**Affiliations:** 1 Department of Chemistry and Biochemistry, Florida International University, Miami, FL 33199, USA; 2 Biomolecular Sciences Institute, Florida International University, 11200 SW 8 St, Miami, FL 33199, USA; 3 Structural Biology Center, X-ray Science Division, Advanced Photon Source, Argonne National Laboratory, 9700 S. Cass Avenue, Lemont, IL 60439, USA; 4 X-ray Science Division, Advanced Photon Source, Argonne National Laboratory, 9700 S. Cass Avenue, Lemont, IL 60439, USA

## Abstract

Type IA topoisomerases interact with G-strand and T-strand ssDNA to regulate DNA topology. However, simultaneous binding of two ssDNA segments to a type IA topoisomerase has not been observed previously. We report here the crystal structure of a type IA topoisomerase with ssDNA segments bound in opposite polarity to the N- and C-terminal domains. Titration of small ssDNA oligonucleotides to *Mycobacterium smegmatis* topoisomerase I with progressive C-terminal deletions showed that the C-terminal region has higher affinity for ssDNA than the N-terminal active site. This allows the C-terminal domains to capture one strand of underwound negatively supercoiled DNA substrate first and position the N-terminal domains to bind and cleave the opposite strand in the relaxation reaction. Efficiency of negative supercoiling relaxation increases with the number of domains that bind ssDNA primarily with conserved aromatic residues and possibly with assistance from polar/basic residues. A comparison of bacterial topoisomerase I structures showed that a conserved transesterification unit (N-terminal toroid structure) for cutting and rejoining of a ssDNA strand can be combined with two different types of C-terminal ssDNA binding domains to form diverse bacterial topoisomerase I enzymes that are highly efficient in their physiological role of preventing excess negative supercoiling in the genome.

## INTRODUCTION

DNA topoisomerases are required for regulating DNA supercoiling and resolving topological entanglements that arise during essential cellular processes including replication, transcription, recombination and repair (1–3). To carry out these catalytic functions, topoisomerases utilize an active site tyrosine nucleophile (4) to break the DNA phosphodiester linkage, form a covalent topoisomerase-DNA intermediate, and allow DNA passage through the break (called the DNA ‘gate’) before the active site tyrosine is regenerated in the DNA rejoining step. Type I topoisomerases break and rejoin one strand of DNA at a time, while type II topoisomerases have two subunits that can break and rejoin the two strands of a DNA duplex (2,5). Type I and type II topoisomerases are further divided into subfamilies based on sequence and mechanism. The defining characteristics of type IA subfamily of topoisomerases include the formation of a 5′-phosphotyrosine bond in DNA cleavage, the requirement of divalent cations, and the selective binding of single-stranded DNA (ssDNA) during catalysis (6–8). More recently, a variety of type IA topoisomerases from every taxonomic kingdom have been shown to possess RNA topoisomerase activities that may be required for promoting mRNA translation (9,10) and RNAi-induced gene silencing (11).

**Figure 1.**
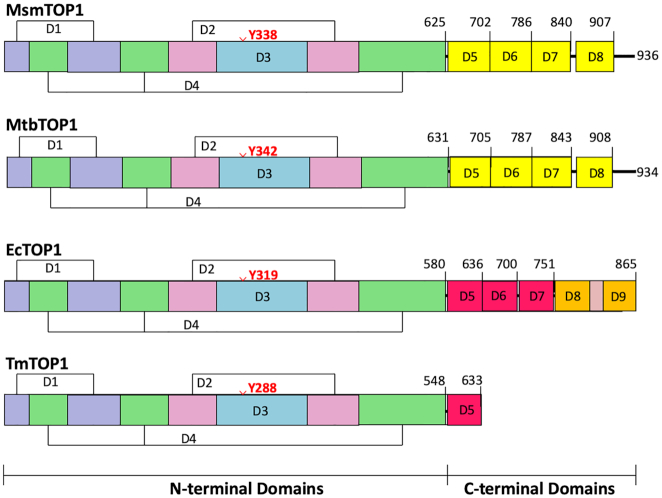
Domain organization in structures of bacterial topoisomerase I. The domain boundaries of MsmTOP1 and MtbTOP1 are based on PDB reported here and PBD 5D5H, with structural homology extended to D8. The EcTOP1 and TmTOP1 domain boundaries are based on PDB 4RUL and PDB 2GAI of the full length structures.

Every bacterium has at least one type IA topoisomerase related to *Escherichia coli* topoisomerase I for overcoming topological barriers that require ssDNA strand passage (12). These bacterial topoisomerase I protein sequences exhibit a high degree of sequence conservation in the N-terminal domains D1–D4, where the active site for the cutting and rejoining of a single DNA strand is located (Figure 1). All structures of type IA topoisomerases available to date show a toroid assembly formed by N-terminal domains D1–D4. However, additional protein sequences in the C-terminal domains are involved in the relaxation of supercoiled DNA. The bacterial *topA* genes that encode topoisomerase I have been classified as segmentally variable genes (13). The variable C-terminal domains of bacterial topoisomerase I have been shown to bind ssDNA with high affinity (14–16). *Escherichia coli* topoisomerase I (EcTOP1) has five Topo_C_ZnRpt repeats, including three zinc-ribbon domains that each bind a Zn^2+^ ion with a tetra-cysteine motif (D5-D7), and two zinc-ribbon like domains (D8, D9) in the C-terminal region (17–20). Different numbers (from 1 to 5) of zinc-ribbon or zinc-ribbon like domains can be found in other bacterial topoisomerase I sequences (21,22). In contrast, topoisomerase I in *Mycobacterium* species and related species of Actinobacteria have Topo_C_Rpt type repeated units in their C-terminal region (Figure 1) that are different from the zinc-ribbon domain (23,24).

We hypothesize that while the N-terminal domains of bacterial topoisomerase I containing the DNA cleavage active site interact with the G-strand ssDNA to create a break necessary for DNA strand passage, the C-terminal domains also need to interact with the T-strand ssDNA with high affinity. These two strands of ssDNA should correspond to the opposite strands of an underwound duplex DNA. The binding of ssDNA to the C-terminal domains would facilitate the DNA substrate recognition, processivity and rapid relaxation of negatively supercoiled DNA during catalysis. The full length EcTOP1 structure (PDB 4RUL) has ssDNA bound to the C-terminal Topo_C_ZnRpt domains (19). To obtain structural information on the mycobacterial Topo_C_Rpt domains interactions with ssDNA, we attempted to produce novel co-crystals of full length *Mycobacterium tuberculosis* and *Mycobacterium smegmatis* topoisomerase I proteins as well as truncated forms that have more Topo_C_Rpt C-terminal domains than the previously crystalized MtbTOP1-704t (D1-D5). We succeeded in obtaining a new crystal structure of a catalytically active 839-residue form of *M. smegmatis* topoisomerase I (MsmTOP1-839t) in complex with a 25-base ssDNA, MTS2-25. The construct MsmTOP1-839t includes the N-terminal domains (D1–D4) and three of four predicted C-terminal domains (Figure 1). In this crystal structure, we can observe the interactions of two ssDNA segments independently with both the N-terminal active site and the C-terminal Topo_C_Rpt domains that are found in mycobacteria and related species. The structure of this MsmTOP1-839t/MTS2-25 complex and its implications for the mycobacterial topoisomerase catalytic mechanism will be the focus of this paper.

## MATERIALS AND METHODS

### Protein expression and purification

The coding sequence for MsmTOP1 was amplified from the genomic DNA of *M. smegmatis* strain mc2 155. The MsmTOP1 expression plasmid was constructed via Gibson Assembly (25) by inserting the MsmTOP1 gene into a pET-His6-Mocr TEV-LIC cloning vector (2O-T) gifted by Scott Gradia (Addgene plasmid #29710). 2O-T expression plasmid for *M. tuberculosis* topoisomerase I (MtbTOP1) and MtbTOP1-704t were constructed as described (23). The NEBbuilder HIFI DNA Assembly kit (New England BioLabs) was used to insert the coding sequence for MsmTOP1-909t and MtbTOP1-910t into the 2O-T vector. The expression clones for MsmTOP1-839t, MsmTOP1-785t, MsmTOP1-701t, MtbTOP1-840t and MtbTOP1-786t were made by site-directed mutagenesis using Q5 High Fidelity DNA polymerase (New England BioLabs) to place a stop codon substitution at the appropriate position in the 2O-T MsmTOP1 and 2O-T MtbTOP1 plasmids. The primers (from Sigma Genosys) used for construction of the expression clones are listed in Supplementary Table S1. The number in each truncation mutant construct corresponds to the last residue before the stop codon for the termination of protein synthesis.

MsmTOP1 and its mutants with different lengths of C-terminal truncations were overexpressed in *E. coli* BL21 star (DE3) strain (Invitrogen) while MtbTOP1and its mutants were overexpressed in *E. coli* T7 Express Crystal strain (New England BioLabs). Cells were first cultured in Luria broth from 1:100 dilution of the overnight cultures at 30°C until exponential phase (OD_600_ = 0.4). Following addition of 1 mM IPTG to induce overexpression, growth was continued at 22°C overnight. Protein purification was carried out as previously described (23,26) with modifications. Briefly, the pelleted cells were lyzed by 3 cycles of freezing and thawing in lysis buffer (50 mM sodium phosphate, 0.3 M NaCl, 10 mM imidazole, pH 8.0, 1 mg/ml lysozyme). Following centrifugation of the lysate, the soluble fraction was mixed with Ni Sepharose 6 Fast Flow (GE Healthcare) at 4°C for 1 h before transfer into a column. After washing with wash buffer (50 mM sodium phosphate, 0.3 M NaCl, 20 mM imidazole, pH 8.0), the His-Mocr tagged recombinant protein was eluted with elution buffer (50 mM sodium phosphate pH 8.0, 0.3 M NaCl, 400 mM imidazole) and dialyzed into TEV reaction buffer (50 mM Tris–HCl, 0.5 mM EDTA and 1mM DTT, pH 8.0) before digestion at 20°C for 6 h with TEV protease for tag cleavage. The digestion reaction was left at 4°C overnight. Ni-Sepharose 6 Fast Flow beads were used to remove the cleaved N-terminal His6-Mocr tag and His-tagged TEV protease. The recombinant proteins were concentrated using the EMD Millipore Ultra Centrifugal Filters with 30 000 Daltons Nominal Molecular Weight Limit (NMWL) to ∼10 ml before loading onto the size exclusion chromatography column HiPrep™ 26/60 Sephacryl S-200 (GE Healthcare Life Sciences). The purified topoisomerase proteins were eluted with column buffer (20 mM Tris–HCl, 0.3 M KCl, pH 8.0) and either concentrated immediately for crystallization or dialyzed against storage buffer (0.1 M potassium phosphate, 0.2 mM EDTA pH 8.0, 50% glycerol) for enzymatic analysis. The protein concentrations were determined with the Bio-Rad Bradford protein assay kit using a bovine serum albumin (Bio-Rad) standard. SDS-PAGE analysis of the purified truncated mutant proteins is shown in Supplementary Figure S1.

### Crystallization

The purified MsmTOP1-839t protein (92.3 kDa) was concentrated to about 38.8 mg/ml (∼0.43 mM) for crystallization. For the co-crystallization trials with ssDNA, the protein was first mixed with ssDNA MTS2-25 (Table 1) in a 1:2 molar ratio and then incubated on ice for 2 h before crystallization set-up. Screening for crystallization conditions was set up with a Mosquito nanoliter liquid handler (TTP LabTech) using the sitting drop vapor diffusion technique in 96-well CrystalQuick plates (Greiner). For each condition, 0.2 μl of MsmTOP1-839t/MTS2-25 and 0.2 μl of crystallization formulation were mixed; the mixture was equilibrated against 140 μl of the crystallization solution in each reservoir well. The crystallization screens used were MCSG-1–4 at 16°C. Crystals appeared under several conditions including crystals under the condition of 0.1 M Tris–HCl pH 8.5 and 1.5 M lithium sulfate. One of the crystals from this condition diffracted to the highest resolution limit (3.1 Å) as described below. For the preparation of the crystals for X-ray diffraction experiments, they were harvested and transferred to cryoprotectant solution that contains 25% glycerol in addition to crystallization buffer for a few minutes and then cryocooled directly in liquid nitrogen.

**Table 1.** Oligonucleotide substrates for crystallization and activity assays

	Length	Sequence
STS32	32	5′-CAGTGAGCGAGCTTCCGC↓TTGACATCCCAATA-3′
MTS2-25	25	5′-CAGTGAGCGAGCTTCCGC↓TTGACTT-3′
MTS3-14	14	5′-CTTCCGC↓TTGACAT-3′
MTS2-14	14	5′-CTTCCGC↓TTGACTT-3′
MTS2-13	13	5′-TTCCGC↓TTGACTT-3′

Cleavage position indicated by ↓ was mapped for MtbTOP1 in previous study (26).

### Data collection and structure determination

Single-wavelength X-ray diffraction data were collected at 100 K from the cryocooled crystals. All data were obtained at the 19-ID beamline of the Structural Biology Center at the Advanced Photon Source at Argonne National Laboratory using the program SBCcollect (27). The intensities of each data set were integrated, scaled, and merged with the HKL-3000 program suite (28). The data set with the highest resolution limit (3.1 Å) was used for the following structural determination and refinement. The structure of MsmTOP1-839t/MTS2-25 was determined using the molecular replacement method (29). The holo form of MtbTOP1-704t/MTS2-11 (PDB code: 6CQI) (26) that includes the N-terminal toroidal assembly (D1-D4 domains) and the first C-terminal domain (D5) of MtbTOP1-704t was used as a search template. From the symmetry and the dimension of the crystal unit cell, at least two MsmTOP1-839t molecules were expected within one asymmetric unit. After running the rotation and translation functions, the positions of two MsmTOP1 (D1-D5) were easily located. In the difference Fourier maps, extra electron densities associated with the DNA-binding site at the N-terminal domains indicated a similar DNA-binding mode as observed in the MtbTOP1-704t /MTS2-11 structure (26). The following model rebuild, including building one ssDNA into each of the two MsmTOP1 (D1-D5) models, was performed using the program Coot (30). After several alternate cycles of model building and refinement, each of the two initial DNA-binding MsmTOP1 (D1-D5) models was expanded to include the C-terminal D6-D7 domains and the rest of nucleotides in MTS2-25. The final model was refined using the program phenix.refine (31) (Table 2).

**Table 2.** Data collection and refinement statistics

Data collection	MsmTOP1-839t/MTS2-25
Space group	*P*2_1_2_1_2_1_
Unit cell dimensions	
*a, b*, c (Å)	133.3, 135.1, 145.1
*α, β, γ* (°)	90, 90, 90
Protein MW Da (residue)	92 202 (839)^a^
DNA MW Da (residue)	7 649 (25)
Mol or complex (AU)	2
Wavelength (Å)	0.9793
Resolution (Å)	3.11–47.4
Number of unique reflections	47 334 (2 351)^b^
Completeness (%)	98.9 (99.5)^b^
Redundancy	4.9 (5.4)^b^
*R*_merge_	0.073 (0.849)^b^
*I*/σ(*I*)	29.9 (1.7)^b^
CC_1/2_	(0.764)^b^
Wilson B-factors (Å^2^)	110.0
***Phasing*** ^c^	
Correlation coefficient	0.668
***Refinement***	
Resolution (Å)	3.11–47.4
Number of unique reflections (work/test)	44 960/2 282
*R*_work_/*R*_free_ (%)	22.5/27.5
No.of atoms	
Protein/DNA	11 351/984
Water/others	0/110
*B*-factors	
Protein/DNA (Å^2^)	142.3/135.0
Water/others (Å^2^)	NA/189.2
R.m.s, deviation	
Bond length (Å)	0.002
Bond angle (°)	0.443
Ramachandran plot (%)	
Preferred regions (%)	92.9
Allowed regions (%)	6.69
Outliers (%)	0.39
**PDB code**	6PCM

^a^Not including three N-terminal vector-derived residues, SNA.

^b^Last resolution bin, 3.11–3.15 Å.

^c^Molecular replacement (29).

### Relaxation activity assay

Wild-type and mutant topoisomerase proteins were incubated with 250 ng negatively supercoiled plasmid pBAD/Thio (purified by CsCl density gradient centrifugation) in a volume of 20 μl with 10 mM Tris–HCl (pH 8.0), 50 mM NaCl, 0.1 mg/ml gelatin and 5 mM MgCl_2_ at 37°C for 30 min. The reactions were terminated by the addition of 4 μl of stop solution (50 mM EDTA, 50% glycerol and 0.5% (v/v) bromophenol blue). The DNA was then electrophoresed in 1% agarose with TAE buffer (40 mM Tris-acetate, pH 8.0, 2 mM EDTA). The gels were stained with 1 μg/ml concentration ethidium bromide for 1 h and photographed over UV light.

### DNA cleavage assay

The single-stranded oligonucleotide substrates (Table 1) were radiolabeled with γ-^32^P-ATP at the 5′ end using T4 polynucleotide kinase (New England BioLabs). Serial dilutions of wild-type or mutant topoisomerase was incubated with 100 nM or the indicated amount of ^32^P-labeled oligonucleotide substrate in 5μl of 10 mM Tris–HCl (pH 8.0) at 37°C for 30 min. An equal volume of loading solution (79% formamide, 0.2 M NaOH, 0.04% bromophenol blue) was added to terminate the cleavage reactions. The reactions were then heated at 95°C for 5 min before electrophoresis in 15% (STS32 and MTS2-25) or 20% (MTS3-14, MTS2-14, MTS2-13) sequencing gel with TBE running buffer (89 mM Tris-borate, 2 mM EDTA pH 8.3). The cleavage products were visualized using the Pharos FX Plus Phosphor-Imager (Bio-Rad) and densitometry analysis was done with Alphaview (Bio-techne) software.

### DNA religation assay

Wild-type and mutant topoisomerase (200 nM) were first incubated with 100 nM 5′-labeled oligonucleotide substrate STS32 in 5 μl of 10 mM Tris–HCl (pH 8.0) at 37°C for 15 min before the addition of 10 mM MgCl_2_ plus 1 M NaCl to shift the cleavage-religation equilibrium towards DNA religation and dissociate the non-covalently bound topoisomerase from the religated oligonucleotide substrate. After 15 min further incubation at 37°C, the reactions were terminated with equal volume of loading solution and heated at 95°C for 5 min. The reaction products were analyzed by electrophoresis in 15% sequencing gel and analyzed by Phosphor-lmager.

### DNA binding assay

Oligonucleotides STS32 or MTS3-14 modified with 6-carboxyfluorescein at the 3 end were supplied by Biosearch Technologies. Anisotropy assay for binding to the full length MsmTOP1 or truncated MsmTOP1-701t was conducted in 50 mM Tris–HCl, pH 7.5, 100 mM NaCl, 0.1 mM EDTA at room temperature. Control experiments were performed by titration with buffer in the same volumes as the proteins added. Anisotropy measurements were made in the Varian Cary Eclipse fluorescence spectrophotometer with excitation wavelength set at 495 nm and emission wavelength set at 520 nm using excitation and emission slits of 5 and 10 nm. The data was analyzed using the GraphPad Prism program as previously described (32).

### Solution biological small-angle X-ray scattering experiment

Solution biological small-angle X-ray scattering (SAXS) experiments were performed at beamline 12-ID-B of the Advanced Photon Source (APS) at the Argonne National Laboratory. The wavelength, λ, of X-ray radiation was set to 0.886 Å. Scattered X-ray intensities were measured using a Pilatus 2M detector. The sample-to-detector distance was set such that the detecting range of momentum transfer *q* [ = 4π sinθ/λ, where 2θ is the scattering angle] was 0.004–0.8 Å^−1^. Samples of MsmTOP1-839t and MsmTOP1-839t/MTS2-25 were measured using a flow cell consisting of a cylindrical quartz capillary 1.5 mm in diameter with a 10 μm wall thickness. The flow rate was set at 10 μl/s and the exposure time was set to ∼1 second to reduce possible radiation damage. In order to obtain good signal-to-noise ratio values, more than forty images were acquired for each sample and background. The 2D scattering images were converted to 1D SAXS (*I*(*q*) versus *q*) curves through azimuthally averaging after solid angle correction and then normalized with the intensity of the transmitted X-ray beam flux, using beamline software. Both the protein/complex samples and the matched buffer were measured. The SAXS profile of the protein was obtained by subtracting the buffer background from the sample data. Every protein/complex sample was measured at a series of concentrations of 1.0, 2.0 and 5.0 mg/ml, and background-subtracted SAXS data were extrapolated to zero concentration to correct the possible structure factor in low *q* region caused by interparticle interaction/concentration effect.

The radius of gyration (*R*_g_) was calculated using the Guinier equation (33), i.e. $I\ ( q ) = \ I_0\ {\rm{exp}}( { - R_{\rm g}^2{q^2}/3} )$, where *I*_0_ is the forward scattering. The radius of gyration values were calculated from the Guinier equation were 45.3 ± 3.0 versus 41.7 ± 2.1 Å for MsmTOP1-839t and MsmTOP1-839t/MTS2-25, respectively. The pair distance distribution function (PDDF, *P*(*r*)) that is the inverse Fourier transform of X-ray scattering data and roughly a weighted histogram of atomic-pair distances in the molecule, was calculated using GNOM v4.6 (34). The largest molecular dimensions (Dmax) estimated from PDDFs were ∼160 and ∼140 Å for MsmTOP1-839t and MsmTOP1-839t/MTS2-25, respectively.

Three-dimensional molecular envelopes were calculated from SAXS data, using DAMMIF (35) with *q* up to *q* of 0.30 Å^−1^, and GASBOR v2.3i (36) with *q* up to *q* of 0.50 Å^−1^. In 3D molecular envelope reconstructions, the programs use simulated annealing algrithom to search structures that match the SAXS data. Twenty runs were performed for MsmTOP1-839t and MsmTOP1-839t/MTS2-25, respectively. The reconstructions were aligned, averaged, and filtered on the basis of occupancy to generate a consensus structural model using DAMAVER v5.0 (37). The consensus models and individual models were displayed in Supplementary Figures S2 and S3. Similar SAXS envelopes were obtained from DAMMIF and GASBOR calculations. Only the GASBOR calculations were reported here.

## RESULTS

### Crystallization of full-length type IA mycobacterial topoisomerases and their truncated mutants

After determining the crystal structures of MtbTOP1-704t in the absence and in the presence of ssDNA (23,26), we have tried to crystallize full-length mycobacterial topoisomerases in order to further verify our earlier prediction of the C-terminal domain organization of these enzymes and, most importantly, to understand how these distinct C-terminal domains interact with DNA. However, we have failed to crystallize full-length *M. tuberculosis or M. smegmatis* topoisomerases by themselves. Meanwhile, co-crystallization with ssDNA yielded crystals from several conditions. Unfortunately, all of them diffracted poorly. Considering a possible 13-residue flexible link between domain 7 (D7) and domain 8 (D8) of these two mycobacterial topoisomerases (Figure 1), we made two truncated forms terminating after D7, MsmTOP1-839t and MtbTOP1-840t. Both constructs readily crystallized with ssDNA and the X-ray diffraction of these crystals could be improved to ∼3.5 Å resolution. Only one MsmTOP1-839t/MTS2-25 co-crystal diffracted to 3.1 Å. The structure of this MsmTOP1-839t/MTS2-25 complex is reported here.

### Overall crystal structure of MsmTOP1-839t/MTS2-25

**Figure 2.**
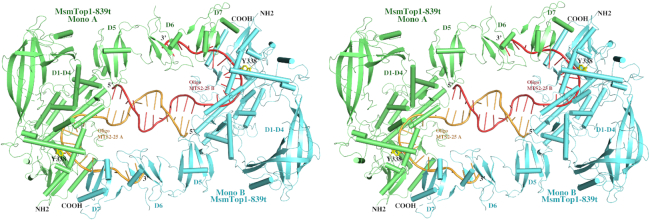
A stereoview of a cartoon diagram of the overall structure of dimer-like MsmTOP1-839t in complex with oligonucleotide MTS2-25. The two MsmTOP1-839t monomers (Mono A and Mono B) are colored in green and cyan, respectively. Their bound MTS2-25 oligonucleotides (MTS2-25 A and MTS2-25 B) are colored in orange and salmon, respectively. The 5′-end regions of the two oligonucleotides primarily mediate the dimerization by forming a 12-base-pair duplex DNA containing six non-Watson–Crick base pairings and mismatches. The 3′-end of the oligonucleotide in one MsmTOP1-839t/MTS2-25 complex interacts with the D6-D7 domains of the other MsmTOP1-839t/MTS2-25 complex. As a result, the N-terminal and C-terminal domains of each MsmTOP1-839t are bound to two opposite strands of an underwound DNA duplex.

There are two MsmTOP1-839t monomers in one asymmetric unit as shown in Figure 2 and Supplementary Figure S4A. In addition to the N-terminal toroidal assembly (D1–D4 domains) and the first C-terminal domain (D5) that have been characterized in MtbTOP1-704t structures, the second and the third C-terminal domain (D6 and D7) have also been resolved (Figure 2). The ∼20-residue insertion between β3- and β4-strands of D6 is disordered with only a few smeared electron density peaks in its expected location even at 0.5σ contour level in Fourier difference maps. The DNA binding specificity at the active site observed in the MtbTOP1-704t/ssDNA structures (26) is maintained with a cytosine nucleotide positioned at the –4 position relative to the expected DNA cleavage site, as seen also in the *E. coli* topoisomerase I covalent complex (38). Within the structure, the 5′-end regions of the two MTS2-25 oligonucleotides in opposite polarity form a 12-base-pair dsDNA containing six non-Watson–Crick base pairings and mismatches (Figure 2, Supplementary Figure S4A). We speculate that the formation of the duplex DNA with partial base-pairing could be a consequence of high concentrations of both protein and ssDNA oligonucleotide used under the given crystallization condition. The duplex DNA structure contributes to the formation of a dimer-like assembly of the MsmTOP1-839t/MTS2-25 complex. As presented in the SAXS experimental results in the following section, the MsmTOP1-839t/MTS2-25 complex is monomeric in solution. In the dimer-like MsmTOP1-839t/MTS2-25 assembly, the C-terminal D6 and D7 domains of MsmTOP1-839t from one MsmTOP1-839t/MTS2-25 complex binds to the very 3′-end of MTS2-25 (5′-TTGACTT-3′) from another MsmTOP1-839t/MTS2-25 complex, and vice versa. Each ssDMA binds to two MsmTOP1-839t monomer simultaneously in forming the molecular packing under crystallization condition. This cross-interaction apparently enhances the dimer-like MsmTOP1-839t/MTS2-25 assembly formation in crystal. More importantly, it shed lights on the way that C-terminal domains of mycobacterial topoisomerases may recognize and act on ssDNA regions in underwound negatively supercoiled DNA (Supplementary Figure S4B).

**Figure 3.**
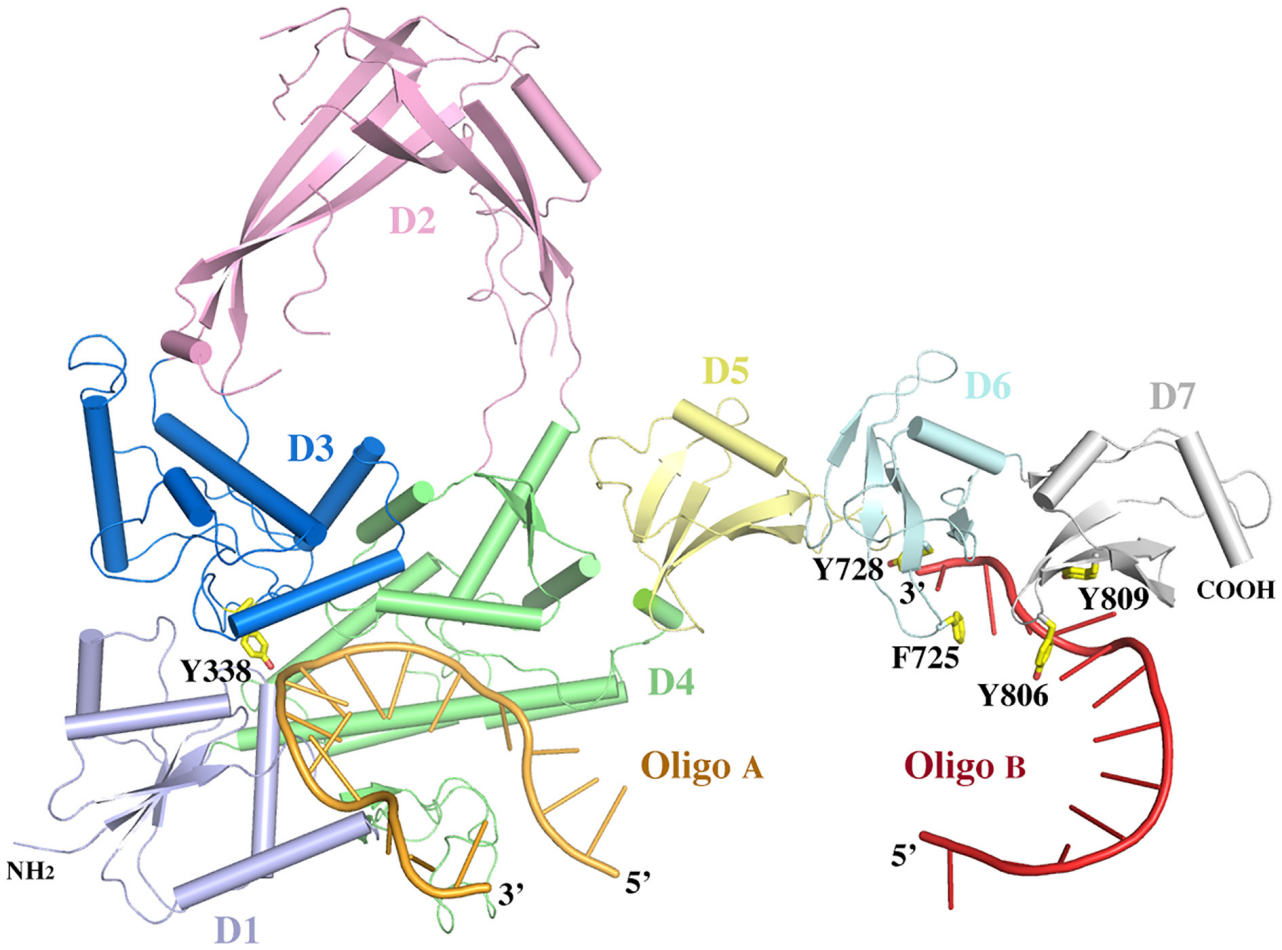
A cartoon diagram of MsmTOP1-839t interacting with ssDNAs in its N- and C-terminal regions. The diagram is adapted from the dimer-like MsmTOP1-839t/MTS2-25 assemble shown in Figure 2. All domains (D1–D7) of MsmTOP1-839t are colored differently. The linker between D4 and D5 is flexible.

In the dimer-like MsmTOP1-839t/MTS2-25 assembly, the two MsmTOP1-839t monomers are very similar to each other. Their structural superposition yields a rmsd (root-mean-square deviation) value of 1.39 Å with the major derivations coming from different tiltings of the N-terminal arch D2 and the C-terminal D6 and D7. No metal ion can be observed at the active site in either monomer (26). Considering that monomer A is more ordered than the monomer B, in the following description and discussion, we will use only the monomer A and its two bound ssDNAs, obtained after removing the dsDNA segment and monomer B, to represent a pre-transition state of MsmTOP1-839t/DNA (Figure 3, Supplementary Figure S4B) that binds ssDNA segments with its N-terminal and C-terminal domains, respectively. In this pre-transition state, the active site tyrosine is in position to attack the scissile phosphate that is four nucleotides downstream of a cytosine recognized in the –4 position by a set of conserved residues (39,40). The different polarity of the two ssDNA segments bound to MsmTOP1-839t can be seen clearly in the stereo view of Figure 2.

### N-terminal domains of MsmTOP1-839t

The N-terminal toroidal assembly (including D1-D4) of MsmTOP1-839t binds ssDNA in the same mode (Figure 3) as that of MtbTOP1-704t that we reported earlier. This is expected, considering the sequence identity (81.1%) between these two mycobacterial type IA topoisomerases. A structural alignment of MtbTOP1-704t to MsmTOP1-839t results in a rmsd value of 1.58 Å for the five domains, D1–D5 (Supplementary Figure S5). If the arch-like D2 from each molecule is not included in alignment, the rmsd value is 1.12 Å, suggesting that D2 contributes to the major deviation between two molecules within D1–D5 region. If the C-terminal D5 is further removed from alignment, the rmsd value is reduced only slightly to 1.02 Å. It indicates that upon ssDNA binding at the N-terminal active site region, the conformation of MsmTOP1 and MtbTOP1 in the region plus D5 is almost the same except for D2. Unlike the other domains in the N-terminal region, D2 is not involved in interdomain protein-protein interactions, and is likely to confer the flexibility needed for opening the DNA gate following ssDNA cleavage by type IA topoisomerases at the active site (41). However, it cannot be ruled out that the presence of the additional C-terminal domains D6 and D7 with bound ssDNA could have indirect influence on the conformation of D2 in the N-terminal region.

### C-terminal domains of MsmTOP1-839t

**Figure 4.**
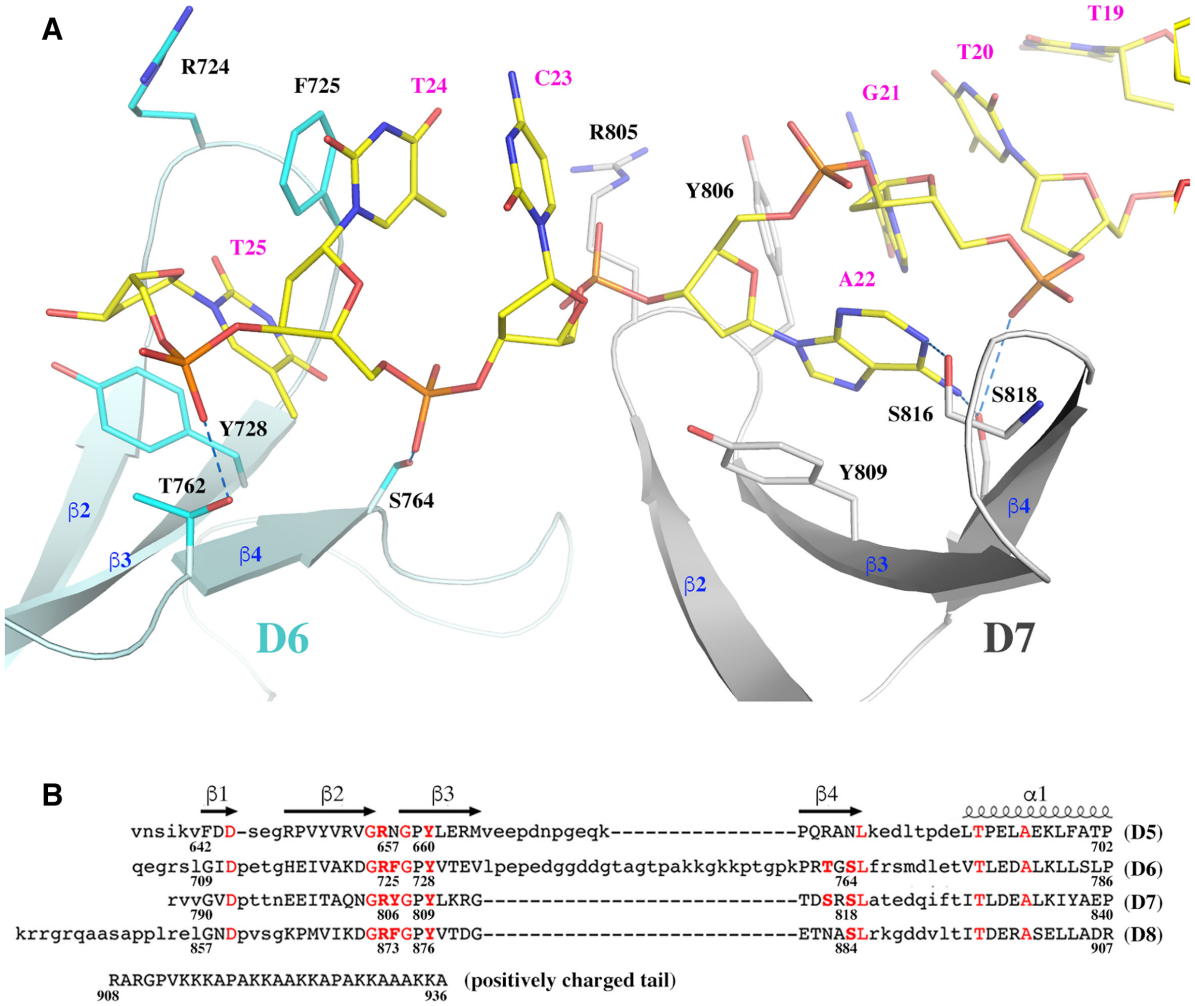
The interaction of the C-terminal domains of MsmTOP1 with ssDNA. (**A**) The interaction of D6 and D7 domains of MsmTOP1 with a ssDNA. The D6 and D7 are colored in cyan and grey respectively. Their key residues involved in DNA bindings are drawn in stick format. (**B**) A structure-based sequence alignment of the four C-terminal domains of MsmTOP1. It is likely that the missing aromatic residue at the β2_ β3 turn in D5 will significantly reduce the binding of D5 to ssDNA.

As predicted earlier (23), the C-terminal domains D6 and D7 resolved in this study have Topo_C_Rpt folds similar to D5 with an antiparallel four-stranded β-sheet flanked by a C-terminal helix on one side (Figures 3 and 4A). Pairwise alignments of the three C-terminal domains suggests that D6 is relatively close to D7 with a rmsd value of 1.45 Å, while D5 is relatively distinct from either D6 or D7 with rmsd values of 2.32 and 2.10 Å, respectively. Their relative structural similarities are consistent with their relative sequence identities (Figure 4B). The sequence identities for the pairs, D5 to D6, D5 to D7, and D6 to D7 are 27.5%, 23.5% and 43.1%, respectively. Based on the sequence identities of D8 to D5 (23.5%), D6 (41.2%) and D7 (35.3%), we predict that the structure of D8 will be more similar to D6 and D7 while more distinct from D5.

The C-terminal domains are generally characterized by a short β1 strand and a β-turn between β2 strand and β3 strand. Two key residues, R and Y/F, are located on the β-turn (Figures 3 and 4A). It is notable that these two key residues are flanked by two glycine residues, which would provide some flexibility for the two key DNA-binding residues. On the β3 strand, there is a highly conserved aromatic residue in both Topo_C_ZnRpt (19) and Topo_C_Rpt C-terminal domains, which also participate in ssDNA-binding.

### Interaction between C-terminal domains and ssDNA

In the MsmTOP1-839t monomer A structure (Figure 3, Supplementary Figure S4B), the C-terminal D6 and D7 bind a ssDNA segment in a 3′ to 5′ direction. Each domain interacts with two consecutive nucleotides, primarily through two parallel π-π stackings (Figure 4A). One of the π–π interactions forms between the sidechain of the conserved tyrosine from β3 strand and the base of 3′-end nucleotide. The second π–π interaction forms between the sidechain of the aromatic residue (F725 of D6 and Y806 of D7) on the β-turn between β2 and β3 strands and the base of 5′-end nucleotide (Figure 4A). Besides the two π–π stackings, the conserved serine residue at the end of β4 strand (S764 of D6 and S818 of D7) potentially forms a hydrogen bond with the phosphate group of the 5′-end nucleotide (Figure 4A). The S818 of D7 also potentially forms a hydrogen bond with the base of the 5′-end nucleotide. The threonine (T762 of D6) or serine (S816 of D7) on the same β4 strand could also be contributing one more hydrogen bond to either backbone or base of the DNA. We do not observe here any interactions of the arginine residue on the β-turn (R724 of D6 and R805 of D7) to form salt bridges to phosphate groups of ssDNA or contribute a hydrogen bond. However, it is not possible to see such interactions for R724 in this structure because the ssDNA has ended at that position. From the ssDNA binding patterns of D6 and D7 and the sequence alignment (Figure 4B), it can be predicted that D8 will bind ssDNA in a similar manner. However, the binding of D5 to ssDNA is questionable due to the lack of an aromatic residue on its β-turn between β2 and β3 strands, as well as the lack of threonine/serine residues on its β4 strand (Figure 4B).

### Solution property of MsmTOP1-839t and MsmTOP1-839t/MTS2-25

**Figure 5.**
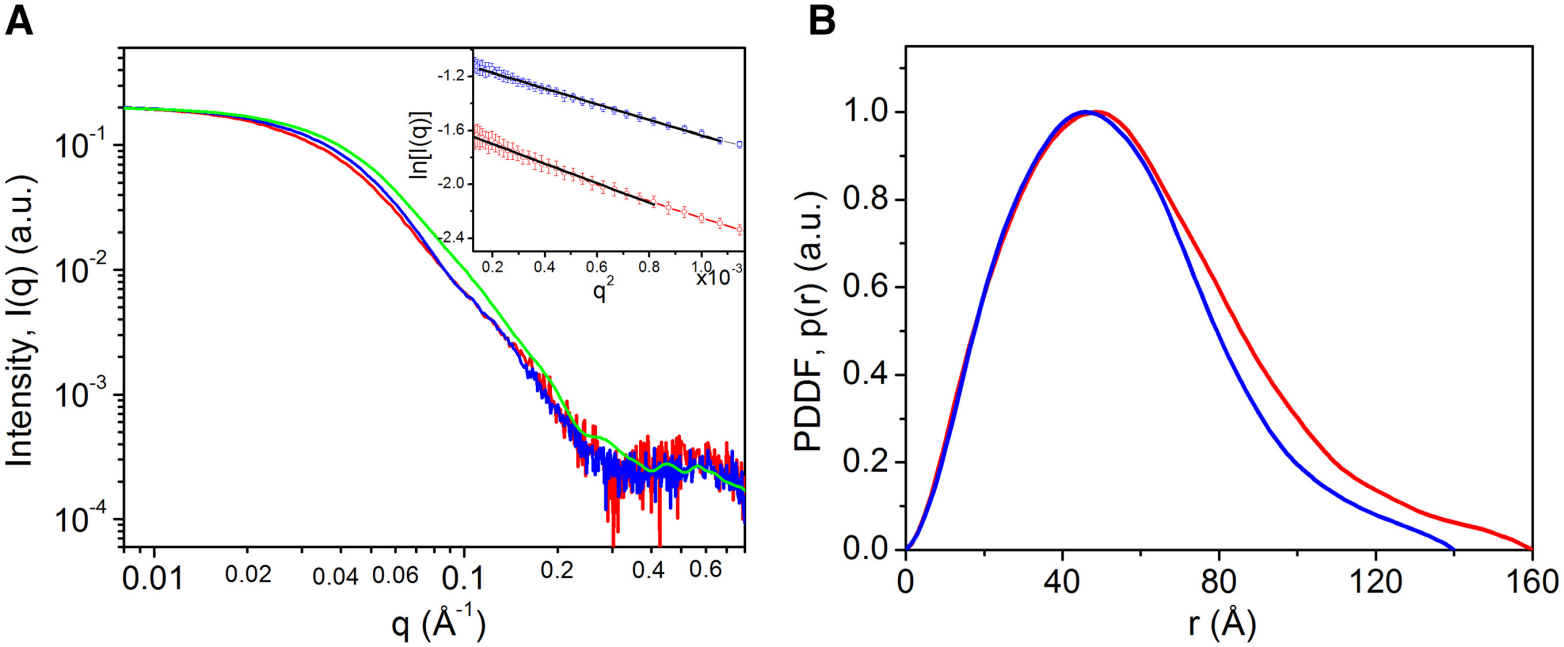
Experimental solution small-angle X-ray scattering (SAXS) data, simulated scattering profiles and pair distance distribution functions derived from SAXS data for MsmTOP1-839t and its complex. (**A**) Normalized experimental SAXS data for MsmTOP1-839t (red) and MsmTOP1-839t/MTS2-25 (blue). Green curve is back-calculated scattering profile for MsmTOP1-839t/MTS2-25 from one monomeric MsmTop1-839t/MTS2-25 of the dimer-like MsmTop1-839Dt/MTS2-25 presented in this paper. Inset: Guinie plots for SAXS data of MsmTOP1-839t (red circles) and MsmTOP1-839t/MTS2-25 (blue squares) and fittings (black lines). The linear Guinier plots indicate the good size homogeneity of those samples. (**B**) Normalized pair distance distribution functions (PDDFs) derived from SAXS data in A: MsmTOP1-839t, red; MsmTOP1-839t/MTS2-25, blue. The r position where *p*(*r*) approaches zero suggests the largest dimension (Dmax) of the molecule. After binding MTS2-25, MsmTOP1-839t becomes slightly more compact.

Solution small-angle X-ray scattering measurements show that MsmTOP1-839t and MsmTOP1-839t/MTS2-25 are monomeric in solution (Figure 5A) and that the dimerization of MsmTOP1-839t/MTS2-25 complex seen in the crystal structure was likely to have resulted from the duplex DNA structure formation by MTS2-25 ssDNA oligonucleotide at high concentration. DNA binding causes an appreciable change in the solution SAXS curves of MsmTOP1-839t, especially in *q* range of 0.01–0.1 Å^−1^, as displayed in Figure 5A. The conformational changes of the toroidal N-terminal region upon ssDNA binding have been reported in both EcTOP1 and MtbTOP1 crystallographic structural studies (26,38). The SAXS data is consistent with similar conformational changes to MsmTOP1 upon DNA binding in solution. However, as the conformational changes for the N-terminal region based on the crystals structures are relatively small, additional conformational changes to C-terminal domains and/or changes in their relative orientations to N-terminal domains, though not resolvable directly from SAXS data, may help account for the considerable conformational change observed in solution here by SAXS.

**Figure 6.**
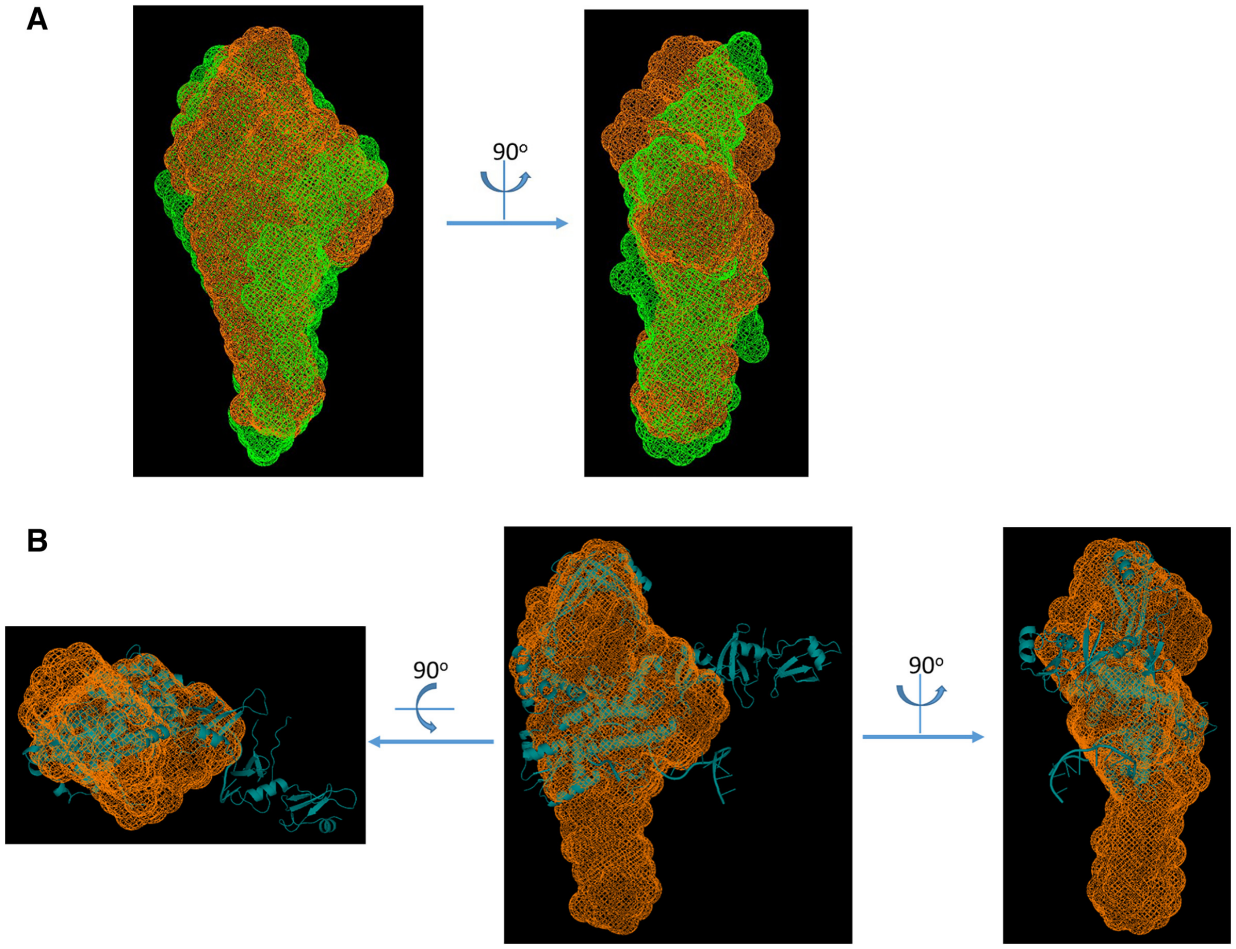
SAXS molecular envelopes and superimposition with atomic structures for MsmTOP1-839t/MTS2-25. (**A**) Two views of overlay of SAXS molecular envelopes for MsmTOP1-839t/MTS2-25 (orange mesh) and MsmTOP1-839t (green mesh). The SAXS envelope of MsmTOP1-839t is slightly longer than that of MsmTOP1-839t/MTS2-25, but both of them are closed, indicating there is no large overall conformational change after MTS2-25 binding, but MsmTOP1-839t becomes more compacted after DNA binding. (**B**) Three views of overlay atomic structure (cyan cartoon) and SAXS envelope (orange mesh) of MsmTOP1-839t/MTS2-25. Compared to the SAXS envelope, the D5–D7 domains of crystal structure of MsmTOP1-839t/MTS2-25 protrude from the sides of the envelope while the bottom part of the SAXS envelope is unfilled. It suggests that in solution the C-terminal D5–D7 domains of MsmTOP1-839t, in either its apo state or its MTS2-25 binding state, may adopt a different orientation relative to the toroidal N-terminal assembly (D1-D4) as observed in crystal structure.

According to the pair distance distribution function shown in Figure 5B, and the molecular envelopes in Figure 6A, both derived from the solution SAXS data, the length of the MsmTOP1-839t/MTS2-25 complex becomes 10–20 Å shorter than MsmTOP1-839t, but both are very similar in SAXS envelope shape. However, the overlay of the SAXS envelope and crystal structure for MsmTOP1-839t/MTS2-25 shows that the C-terminal D5-D7 domains may adopt a different relative orientation to the N-terminal domain in solution with what has been observed in crystal (Figure 6B). Based on the MsmTOP1-839t/MTS2-25 structure, a possible conformation in solution involves the rotation of D5-D7 downward through the flexible linker between D4 and D5 (Supplementary Movie S1, and Supplementary Figure S3). In this way, the structures of either MsmTOP1-839t or MsmTOP1-839t/MTS2-25 will better fit into their SAXS envelopes. The flexibility of the D4–D5 linker would also allow the movement of the ssDNA (T-strand) bound to the C-terminal domains toward the active site of N-terminal domains, passing the gate when the ssDNA (G-strand) bound at the N-terminal domains is cleaved.

### Requirement of MsmTOP1 D6, D7 for relaxation activity

**Figure 7.**
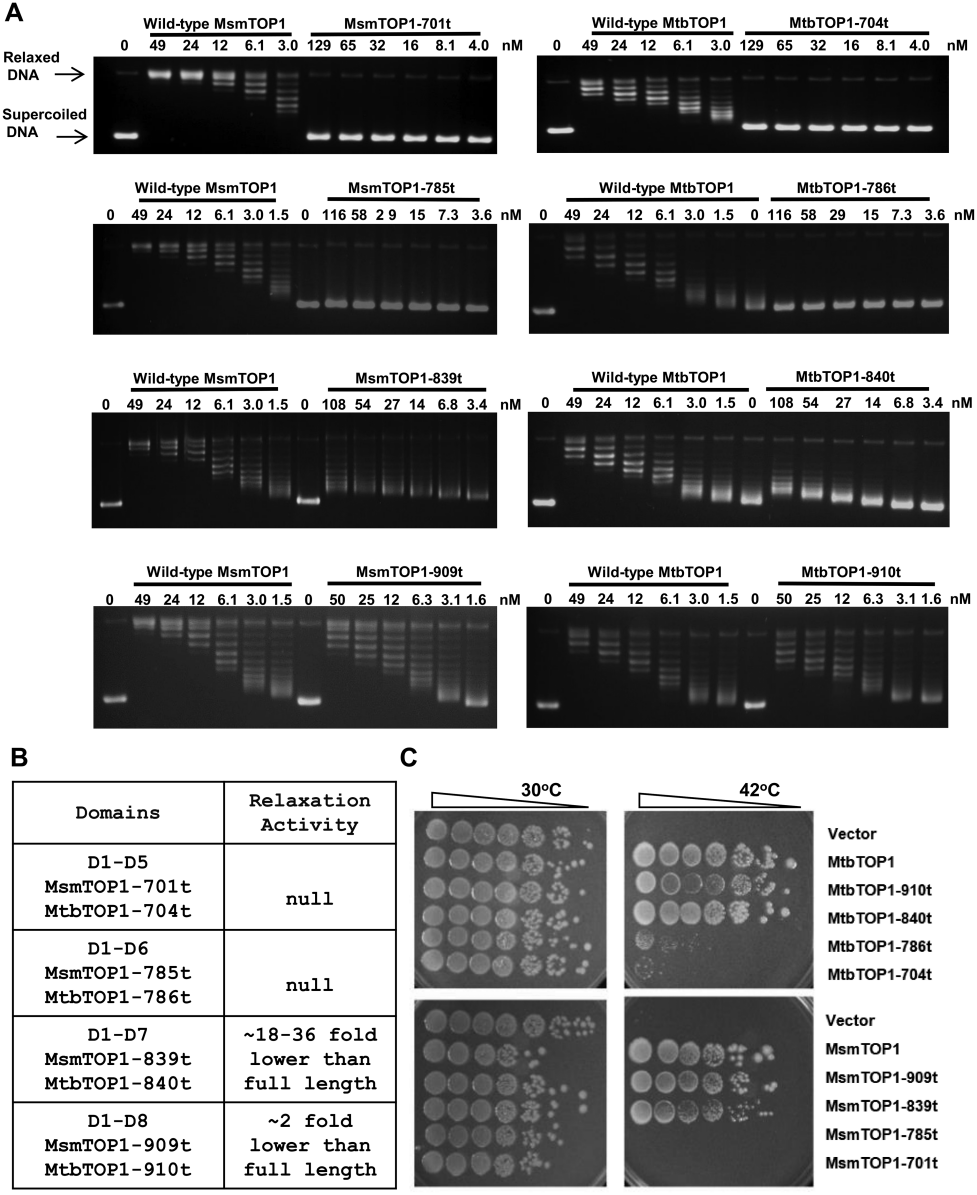
Requirement of C-terminal domains for mycobacterial topoisomerase I relaxation activity. (**A**) Assay of relaxation of negatively supercoiled plasmid DNA by wild-type or mutant MsmTOP1, MtbTOP1 truncated at the end of D5 (MsmTOP1-701t, MtbTOP1-704t), D6 (MsmTOP1-785t, MtbTOP1-786t), D7 (MsmTOP1-839t, MtbTOP1-840t) and D8 (MsmTOP1-909t, MtbTOP1-910t). (**B**) Summary of relaxation activity of the mutants with truncated C-terminal domains. (**C**) Complementation of *E. coli* AS17 with *topA*^ts^ chromosomal mutation for growth at 42°C by wild-type or mutant MtbTOP1 or MsmTOP1 produced by leaky expression from the T7 promoter in the 2O-T cloning vector.

Similar to MtbTOP1-704t, MsmTOP1-701t with D1-D5 only has null relaxation activity when assayed with negatively supercoiled plasmid (Figure 7A and B). MsmTOP1-785t with D1–D6 was also inactive in the assay. Relaxation activity could be observed at level 16–32-fold lower than full length MsmTOP1 for MsmTOP1-839t with D7 also present. Relaxation activity was significantly enhanced by the presence of D8 in MsmTOP1-909t to half the level of full length MsmTOP1 which has an additional 27 residues long basic C-terminal tail. Similar results are obtained from the assays of MtbTOP1-786t (D1–D6), MtbTOP1-840t (D1–D7) and MtbTOP1-910t (D1–D8) relaxation activities (Figure 7A and B). DNA remained only partially relaxed even after prolonged incubation with MsmTOP1-839t (Supplementary Figure S6). Nevertheless, the low level of relaxation activity observed for MsmTOP1-839t and MtbTOP1-840t expressed on the multi-copy plasmid was sufficient for *in vivo* complementation of the temperature sensitive *topA* mutation in *E. coli* AS17 (42,43) for growth at 42°C (Figure 7C). The C-terminal domains are thus necessary for mycobacterial topoisomerase I to increase the linking of negatively supercoiled duplex DNA to relax the superhelical tension, and the efficiency increases with the added numbers of Topo_C_Rpt domains and the presence of the basic C-terminal tail.

### The C-terminal domains of MsmTOP1 and MtbTOP1 have higher binding affinity to ssDNA than the N-terminal domains

**Figure 8.**
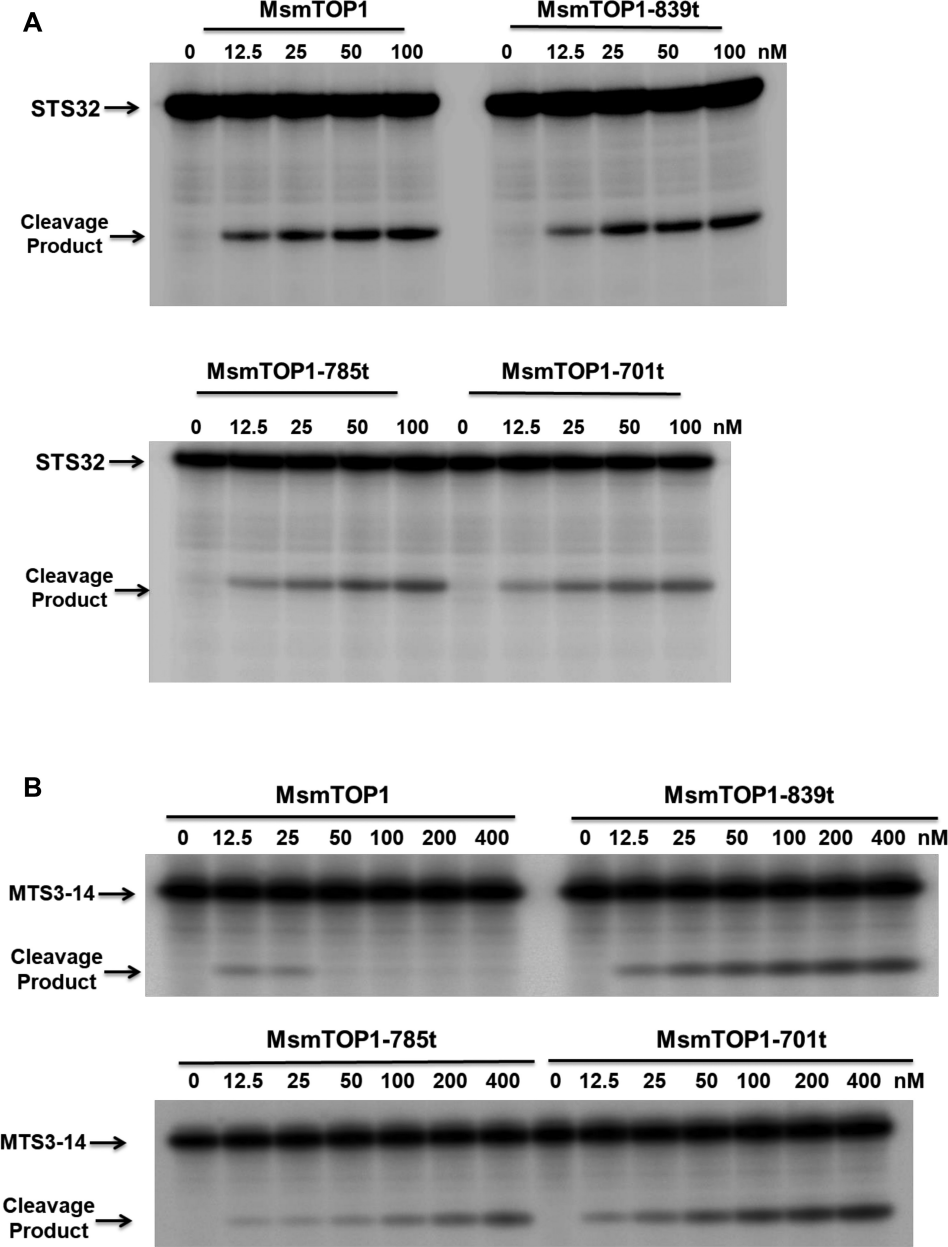
Assay of oligonucleotide cleavage by full length and mutant MsmTOP1 Oligonucleotide substrate was present at 100 nM concentration in each assay. (**A**) Cleavage of 32-base long STS32 oligo increases with increasing enzyme concentration from 0.125:1 to to 1:1 protein–oligonucleotide ratio. (**B**) Cleavage of 14 base long MTS3-14 oligonucleotide by full length MsmTOP1 can be observed at protein-oligonucleotide ratio at 0.125:1 or 0.25:1 but not at higher protein-oligonucleotide ratios.

When we characterized the cleavage of single-stranded oligonucleotide STS32, we observed increase in cleavage product as the protein-oligonucleotide ratios increase for full length MsmTOP1 as well as its mutants with C-terminal domain truncations (Figure 8A). Quantitation of cleavage product formation from three independent experiments is shown in Supplementary Figure S7. The N-terminal domains of MsmTOP1-839t, MsmTOP1-785t and MsmTOP1-701t could also religate the STS32 cleavage product upon addition of Mg^2+^ (Supplementary Figure S8). Interestingly, when a much shorter 14-base long oligonucleotide MTS3-14 was used as cleavage substrate, full length MsmTOP1 yielded cleavage product only at protein-oligonucleotide ratios of 0.125:1 and 0.25:1. At higher protein-oligonucleotide stoichiometries, no cleavage product from MTS3-14 was observed for the full length MsmTOP1 (Figure 8B). This suggests that the short MTS3-14 preferentially binds to the non-reactive C-terminal domains of the full length MsmTOP1. However, when C-terminal domains are truncated, excess protein no longer prevents MTS3-14 binding to the reactive N-terminal domains. Therefore, the cleavage product was observed to increase for MsmTOP1 mutants with C-terminal domain(s) truncated when protein:oligonucleotide ratio is increased from 0.125:1 to 4:1. Similar results have also been observed from MtbTOP1. Full length MtbTOP1 produced cleavage product from STS32 at a 2:1 protein:oligonucleotide ratio, but not from the 13- or 14-base short oligonucleotides MTS3-14, MTS2-14 and MTS2-13; whereas MtbTOP1-704t with C-terminal domain truncation could cleave both STS32 and the short oligonucleotides at the 2:1 protein:oligonucleotide ratios (Supplementary Figure S9).

**Figure 9.**
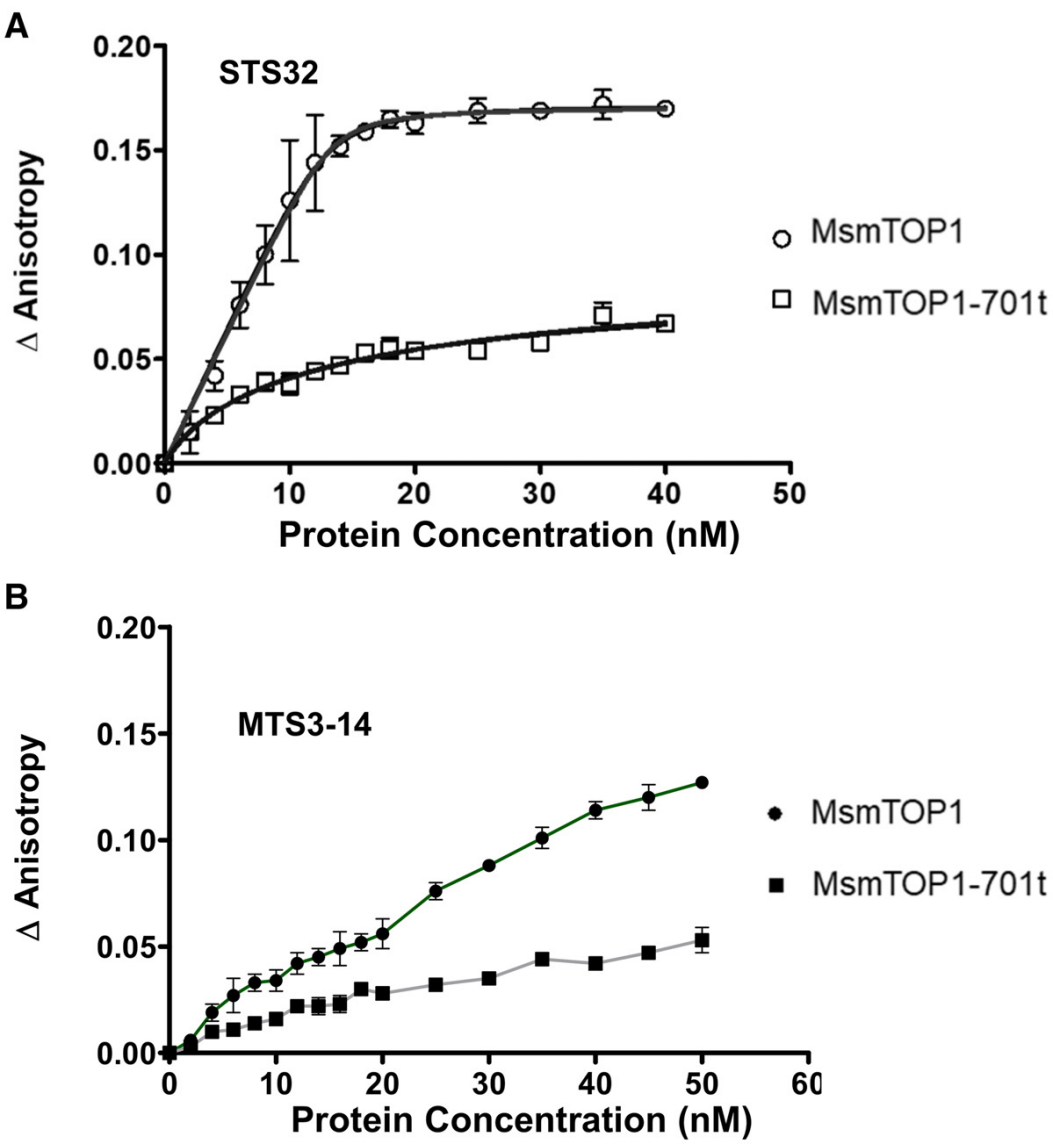
Non-covalent binding of single-stranded oligonucleotides to full length MsmTOP1 and MsmTOP1-701t. Anisotropy assay was used to compare binding of full length MsmTOP1 and MsmTOP1-701t with D1-D5 only to (**A**) STS32 (**B**) MTS3-14 oligonucleotide. Graphs were generated with GraphPad Prism from the change in anisotropy shown here as the average and standard deviation from three experiments.

We used the anisotropy assay to demonstrate that the C-terminal domains of MsmTOP1 provide the major contribution for high affinity binding to ssDNA (Figure 9). The Kd for binding of STS32 to MsmTOP1-701t was measured to be 14.93 nM, versus 0.30 nM for full length MsmTOP1 (Figure 9A). Even though Kds for binding to the 14 base long MTS3-14 substrate could not be determined, the increase in anisotropy upon addition of increasing concentrations of full length MsmTOP1 (Figure 9B) showed that increasing concentrations of the full length enzyme can form a non-covalent complex with MTS3-14, even though cleavage product was not produced at the higher protein:oligonucleotide ratios (Figure 8B). These results indicate ssDNA binds with higher affinity to the C-terminal domains of these mycobacterial topoisomerase I than the N-terminal domains. Therefore, excess full length protein would reduce, or even prevent, binding of MTS3-14 at the N-terminal active site for cleavage to be observed. Meanwhile, the longer STS32 substrate can potentially adopt a U-shaped conformation to permit interaction of two DNA segments in opposite polarity with both the N- and C-terminal domains due to the flexibility of the linker between D4-D5, and also the linkers between the C-terminal domains.

## DISCUSSION

The long term goal of our project is to fully elucidate the mechanism of action of type IA bacterial topoisomerase I. Here we achieved in obtaining a crystal structure of a type IA topoisomerase simultaneously interacting with two ssDNA segments to support the hypothesis that two ssDNA segments can interact with the N- and C-terminal regions of mycobacterial topoisomerase I. The positioning of the preferred cytosine nucleotide in the -4 position (4,38) relative to the scissile phosphate in N-terminal domains indicates that the protein-DNA interactions observed in this crystal structure should correspond to the pre-transition state for DNA cleavage. This is analogous to what was observed in the previously reported MtbTOP1/ssDNA complex structures (26). The structural studies reported here focused on MsmTOP1 because of the availability of the higher quality crystal structure compared to MtbTOP1. We have included MtbTOP1 in the biochemical studies here to demonstrate that the mechanistic role of this specific class of Topo_C_Rpt C-terminal domains is applicable to other mycobacteria. MtbTOP1 is also a validated target for discovery of new TB drugs that may be useful for treatment of MDR TB (44,45).

The ssDNA binding mode with the C-terminal domains in the MsmTOP1-839t/MTS2-25 structure, in comparison to the ssDNA binding mode observed in the EcTOP1 complex structure (19), demonstrates how two different types of structural domains are utilized for ssDNA binding in the C-terminal regions of bacterial type IA topoisomerases (Supplementary Figure S10). The C-terminal domains of MtbTOP1 and MsmTOP1 are representatives of the topoisomerase subgroup with Topo_C_Rpt (23). A domain from the subgroup is typically composed of one four-stranded antiparallel β-sheet, stabilized by a cross-over helix on one side of the β-sheet. On the other hand, the C-terminal domains of EcTOP1 represent the Topo_C_ZnRpt subgroup. A domain from the second subgroup is also composed of one four-stranded antiparallel β-sheet, but is stabilized by a Zn^2+^-binding site formed by four cysteines on the top of each domain. In some cases, the domains in the subgroup maintain the zinc ribbon fold (18), even though they no longer bind Zn^2+^, as shown for D8, D9 of EcTOP1 and the lone C-terminal domain of *Thermotoga maritima* topoisomerase I (22). A common feature of these C-terminal domains of both subgroups is their four-stranded antiparallel β-sheets, which attribute some of their common DNA-binding features (Supplementary Figure S10). From the observation of DNA binding in the C-terminal domains of EcTOP1 and MsmTOP1, an aromatic residue from β3 strand (Y in MtbTOP1/MsmTOP1 and F/Y/W in EcTOP1) plays a key role in ssDNA binding by forming a parallel π–π stacking with the base of one nucleotide. Another aromatic residue that forms the second parallel π–π stacking with the base of a neighboring nucleotide is from the turn between β2 and β3 strands in MtbTOP1/MsmTOP1 or from the turn/loop, either between β2 and β3 strands or between β3 and β4 strands in EcTOP1. Besides the two π–π interactions, certain polar residues (Ser/Thr/Asn), mostly from β4 strand, make hydrogen bonds to DNA. In the EcTOP1 complex structure (19), several arginines interact directly with the ssDNA backbone phosphate groups. In the MsmTOP1-839t/MTS2-32 structure, it is found that the conserved arginine from the turn between the C-terminal domain β2 and β3 strands could potentially interact with ssDNA, although DNA was not present at that position due to the length of the oligonucleotide. We believe these arginines play important roles, at least in the initial steps of ssDNA binding prior to the conformational transition to the structure seen in the MsmTOP1-839t/MTS2-25 complex. Additionally, basic residues in the C-terminal tail that follow the last Topo_C_Rpt domain in MsmTOP1 and Streptomyces topoisomerase I have been shown to play important roles for binding DNA and facilitating the relaxation of negatively supercoiled DNA (15,16).

In contrast to the variable C-terminal domains, the highly conserved N-terminal toroid-forming domains (D1–D4) with the transesterification active site can even be found in the archeal type IA topoisomerases. The 540-residue long topoisomerase I of *Aquifex aeolicus* (Uniprotein ID O66893) shares similar functions with bacterial topoisomerase III enzymes that have relatively short C-terminal region, containing neither Topo_C_Rpt nor Topo_C_ZnRpt domain. The topoisomerase I of *Aquifex aeolicus* may represent the prototype form of type IA topoisomerases. Bacterial topoisomerase I proteins in many phyla have in their C-terminal regions the Topo_C_ZnRpt motif(s) that is a signature of the zinc ribbon domain(s) (20). Meanwhile, C-terminal domains containing Topo_C_Rpt motif(s) are found in topoisomerase I enzymes in the Actinobacteria phylum including the *Mycobacteriaceae* and *Streptomyceae* families, as well as in *Synechococcus elongatus* and *Prochlorococcus marinus* that belong to the Cyanobacteria phylum. There are few studies to understand why each of these topoisomerase I enzymes is associated with one type of C-terminal domain instead of the other. It was proposed that the growth environments encountered by *Mycobacteriaceae* may favor the adaptation of the Topo_C_Rpt domain instead of Topo_C_ZnRpt domain for ssDNA binding by topoisomerase I (16).

A previous study (46) on the structural diversity of supercoiled DNA revealed that supercoiled DNA could assume a wide distribution of three-dimensional conformation. As negative supercoiling increases, bases are increasingly exposed. In the MsmTOP1-839t/MTS2-25 structure, the orientation and distance between the two bound ssDNA segments might be impacted by the molecular packing in crystal, but could still resemble the base-pair separation and helical distortion of parts of a supercoiled DNA under high degree of torsional strain. Similar to an underwound negatively supercoiled DNA, the duplex DNA with partial base-pairing observed in the MsmTOP1-839t/MTS2-25 structure provides each MsmTOP1-839t molecule with two ssDNA segments for binding (Figure 3). Though the crystal structure does not inform the binding sequence of these two ssDNAs, the results from the biochemical experiments reported here suggest that the C-terminal domains bind one ssDNA (T-strand) with a higher affinity than the binding of the second ssDNA (G-strand) to the N-terminal active site for cleavage by the catalytic tyrosine to create the DNA gate. The N-terminal domains interact with eight nucleotides (from –6 nucleotide to +2 nucleotide). In the MsmTOP1-839t/MTS2-25 complex structure, the two C-terminal D6 and D7 bind five nucleotides. Based on the same binding pattern, the C-terminal D6–D8 could bind up to 8 nucleotides. Additionally, MsmTOP1 also has a positively charged insertion within D6, a positively charged linker between D7 and D8, and an extended, highly positively charged tail after D8. The sum of these positively charged structural elements could contribute to the higher DNA binding affinity of the C-terminal domains as previously proposed (16). The prevention of R-loop accumulation associated hypernegative DNA supercoiling is a major physiological function of bacterial topoisomerase I (47). We propose that the higher affinity of the T-strand to the C-terminal domains versus the relatively lower affinity of the G-strand to the N-terminal domains provides a mechanistic control on the sequence of topoisomerase I action for removal of negative supercoils from genomic DNA. Based on the indications for flexibility of the domain arrangement, we speculate that binding and dissociation of ssDNA to the topoisomerase I C-terminal domains should be fast. Initial binding of ssDNA by the C-terminal domains will allow recognition of underwound DNA as T-strand, and provides easy access of the N-terminal domains to the complementary G-strand, so that the cleavage of the G-stand at the active site can be coupled rapidly to the movement of the T-strand to pass through the break for rewinding of the DNA duplex. The mechanism of binding both strands of the DNA duplex before DNA cleavage would also help avoid accidental cleavage of exposed ssDNA by the N-terminal domains, which may lead to a double strand break (48) and genome instability.

It is not yet clear how the binding of the G-strand to N-terminal domains may reposition the C-terminal domains via their flexible linker to bring the T-strand close to active site. We have tried to link the proposed reaction intermediates in order to track the pathways for the conformational changes of the enzyme in its interaction with DNA (see Supplementary Movie S1). The initial conformation of MsmTOP1-839t in the movie was modeled based on SAXS data as described earlier, presumably representing an apo form of the molecule in solution. Its subsequent transition to the conformation seen in the MsmTOP1-839t/MTS2-25 crystal structure is firstly induced by the a higher affinity binding of T-strand to the C-terminal domains. Then it is followed by the relatively lower affinity binding of G-strand to the N-terminal domains. The latter binding causes a significant rearrangement of N-terminal domains. It is not yet clear how the binding of the G-strand to N-terminal domains may change the conformation of the C-terminal domains. We believe the flexibility of the linker between the N-terminal and C-terminal domains of bacterial topoisomerase I is important for the movement of the T-strand bound C-terminal domains toward the G-strand gate opened on N-terminal domains for T-strand passage. We have proposed a possible trajectory for such a movement (Supplementary Movie S1). Additional structural and biochemical studies are needed to elucidate this and all actual conformational changes that occur during the topoisomerase I catalytic cycle.

## DATA AVAILABILITY

Atomic coordinates and structure factors for the reported crystal structure have been deposited with the Protein Data Bank under accession number 6PCM. Dataset files have been uploaded for the referees.

## Supplementary Material

gkaa201_Supplemental_Files
