# Supplementary material for: Mechanistic insights from structure of Mycobacterium smegmatis topoisomerase I with ssDNA bound to both N- and C-terminal domains
Source: Nucleic Acids Res. 2020 Mar 30;48(8):4448–62. doi: 10.1093/nar/gkaa201 (PMC7192597; doi:10.1093/nar/gkaa201)
Supplement: gkaa201_Supplemental_Files [file gkaa201_supplemental_files.zip › Revised Supplementary Information_020420.pdf]

Supplementary Information

Table S1 Sequences of oligonucleotide primers used expression clones construction

Primer name	Primer sequence (5'-3')
MsmTOP1 forward	GGGATCGAGGAAAACCTGTACTTCCAAATGGCTGGCGGCGACCG
MsmTOP1 reverse	GCGGATCCGTTATCCACTTCCAATATTGTTCGGCGGAAAC**<u>CTA</u>**GGCCTTCTT
MsmTOP1-909t forward	GGGATCGAGGAAAACCTGTACTTCCAAATGGCTGGCGGCGACCG
MsmTOP1-909t reverse	GCGGATCCGTTATCCACTTCC**<u>AAT</u>**ATTGTTAGGCACGGCGGTCGG
MsmTOP1-839t forward	CGGCCACGGCGTTT**<u>CTA</u>**CTCGGCGTAGATCTT
MsmTOP1-839t reverse	AAGATCTACGCCGAG**<u>TAG</u>**AAACGCCGTGGCCG
MsmTOP1-785t forward	GCCCACGACGCG**<u>CTA</u>**CAGCGACAGCAGC
MsmTOP1-785t reverse	GCTGCTGTCGCTG**<u>TAG</u>**CGCGTCGTGGGC
MsmTOP1-701t forward	GCGTCCCTCTTG**<u>CTA</u>**TGTGGCGAAGAGCTTTTCGG
MsmTOP1-701t reverse	CCGAAAAGCTCTTCGCCACA**TAG**CAAGAGGGACGC
MtbTOP1-910t forward	GATCGAGGAAAACCTGTACTTCCAATTGGCTGACCCGAAAACGAAGGG
MtbTOP1-910t reverse	GCGGATCCGTTATCCACTTCCAATATTG**<u>CTA</u>**GGCTCGGCGATCGGCCAA
MtbTOP1-840t forward	CCGGCCACGACGTTT**<u>CTA</u>**CTCTGCGTAGATCTTC
MtbTOP1-840t reverse	GAAGATCTACGCAGAG**<u>TAG</u>**AAACGTCGTGGCCGG
MtbTOP1-786t forward	GCTGTCACTG**<u>TAG</u>**CGCGTGGTCG
MtbTOP1-786t reverse	CGACCACGCG**<u>CTA</u>**CAGTGACAGC
MtbTOP1-704t forward	GTCCCTGTTG**<u>CTA</u>**TGTGGCAAAGAGCTCTTCGGC
MtbTOP1-704t reverse	GCCGAAGAGCTCTTTGCCACA**<u>TAG</u>**CAACAGGGAC

The number in each primer corresponds to the last residue before termination of the coding sequence with the termination codon in forward or reverse direction (bold, underlined, in red).

Movie S1: A proposed sequence of conformational changes of MsmTOP1-839t upon its sequential binding to ssDNAs with its C- and N-terminal domains, respectively. The full-length MsmTOP1, which includes additional C-terminal D8 domain and a positively charged tail, is expected to follow a similar conformational change path. The movie illustrates trajectories that morph MsmTOP1-839t from an apo form (Model 1) to a holo form with a ssDNA (T-strand) bounded to its C-terminal domains (Model 2), then to a holo form with the second ssDNA (G-strand) bound to its N-terminal active site (Model 3), finally to a conformation in which C-terminal domains bring T-strand close to the active site (Model 4) for passing the gate after G-strand is cleaved. The C-terminal domains motion together with T-strand in Model 4 is speculative with no supporting data. Select images from the movie are shown here in sequence.

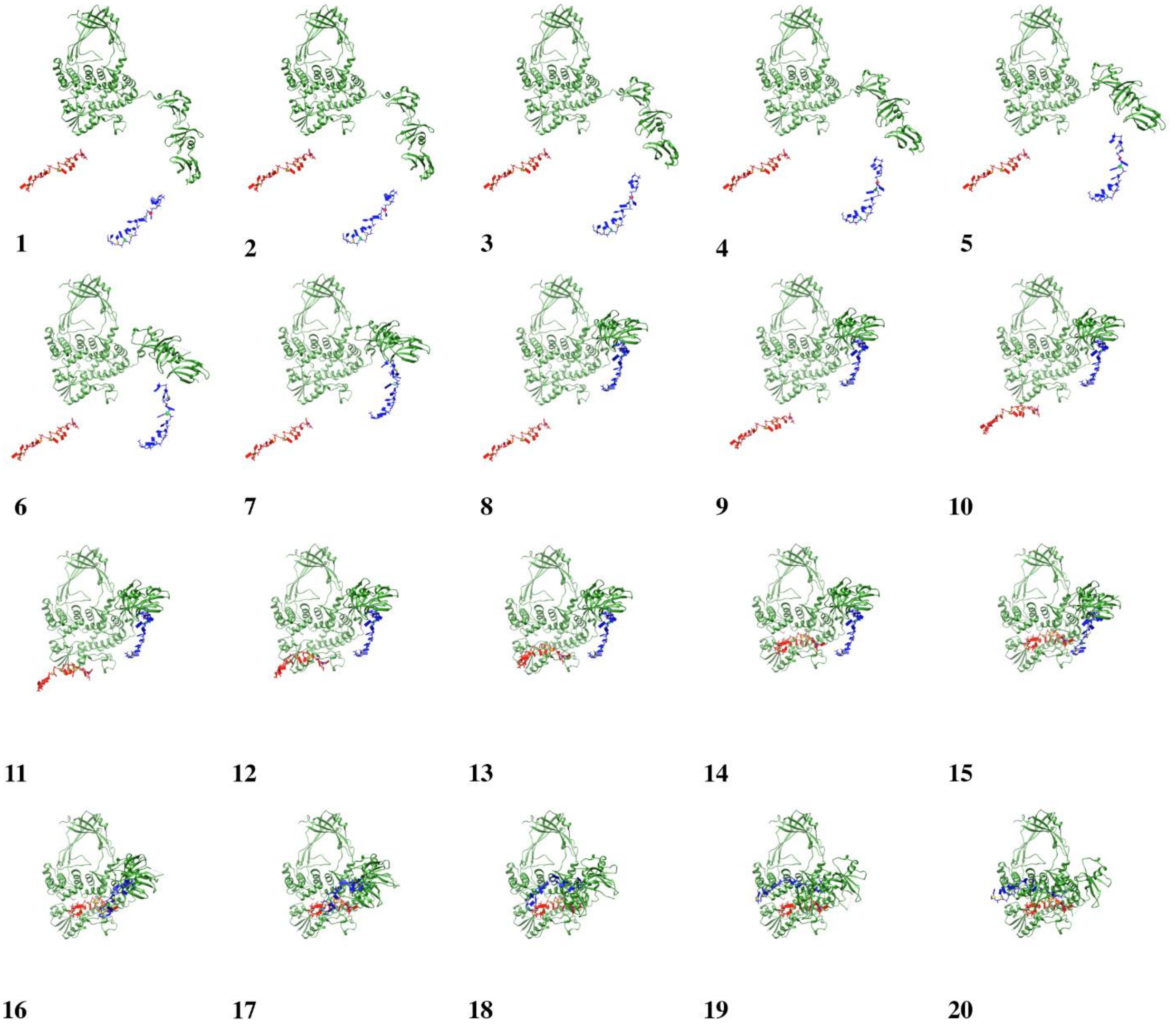

The modeling of the Model 1 is based on the apo form MtbTOP1-704t structure (PDB code: 5UJ1) for its N-terminal domains part and the MsmTop1-839t/MTS2-25 structure (PDB 6PCM reported in this study) for its C-terminal domains part. The relative orientation of the C-terminal domains to the N-terminal domains

of the apo form MsmTOP1-839t is based on the SAXS profile of MsmTOP1-839t in solution as described in main text. The creation of the Model 2 is based on the MsmTOP1-839t/MTS2-25 structure with its N-terminal domains re-modeled based on the apo form MtbTOP1-704t structure (PDB code: 5UJ1). The Model 3 is simply based on the MsmTOP1-839t/MTS2-25 structure. The Model 4 is also based on the MsmTOP1-839t/MTS2-25 structure, but with its C-terminal domains being manually repositioned in order to move the bound T-strand towards to the N-terminal active site. In each model, two ssDNAs represent part of T-strand (in blue) and G-strand (in red), respectively. The morph movie was made with UCSF Chimera program (1).

Reference

1. Pettersen E.F., Goddard T.D., Huang C.C., Couch G.S., Greenblatt D.M., Meng E.C. and Ferrin T.E. (2004) UCSF Chimera—A Visualization System for Exploratory Research and Analysis. *J Comput Chem*. **25**, 1605-12)

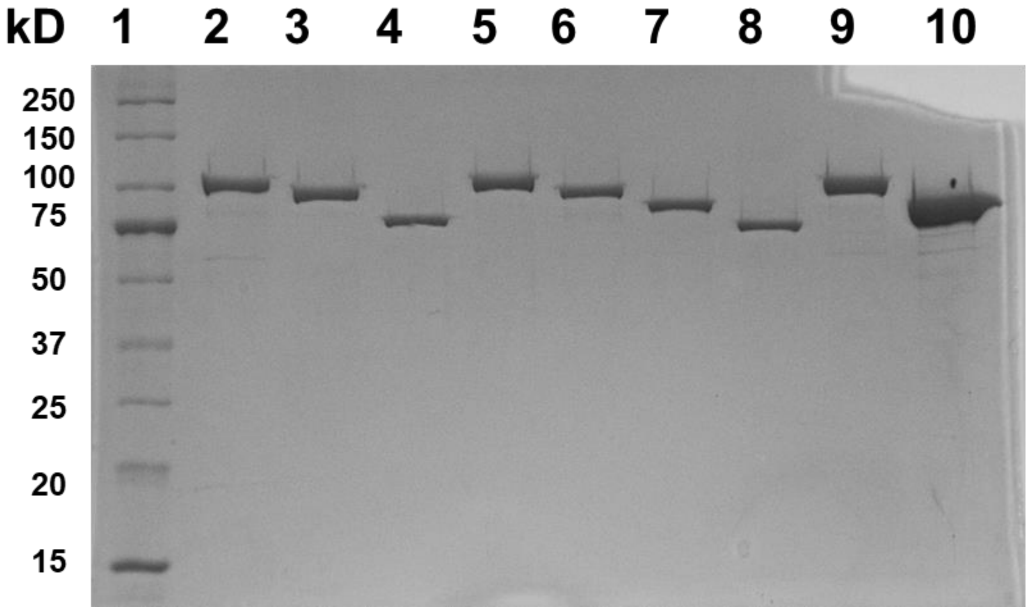

Figure S1. SDS-PAGE analysis of purified recombinant proteins. The proteins were visualized with Coomassie Blue staining following electrophoresis in a 10% SDS gel. Lane 1: Molecular weight standards; Lane 2: MtbTOP1-910t; Lane 3: MtTOP1-840t; Lane 4:MtbTOP1-704t; Lane 5: MsmTOP1-909t; Lane 6: MsmTOP1-839t; Lane 7: MsmTOP1-785t; Lane 8: MsmTOP1-701t; Lane 9: Wild-type MsmTOP1; Lane 10: MtbTOP1-786t

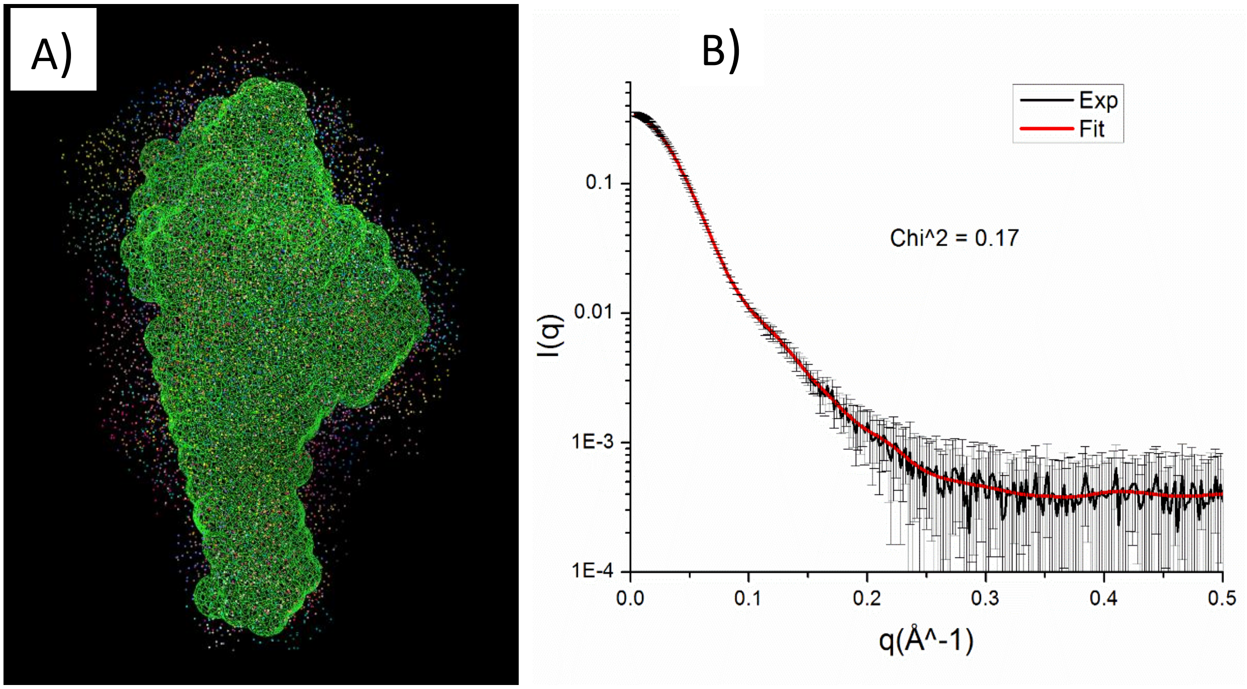

Figure S2. SAXS GASBOR model calculations for MsmTOP1-839t/MTS2-25 (**A**) Individual ab initio models (dots, each color represents one structural model) calculated using program GASBOR and averaged model (green mesh) for MsmTOP1-839/MTS2-25. In GASBOR calculations, 2.5 dummy amino acid residues were used to account for x-ray scattering contribution of 1 nucleotide. Program DAMAVER was used to average those individual models after removing "water molecules" in each individual GASBOR model. The normalized spatial discrepancy (NSD) of those models is: NSD = 1.8 ± 0.1, which is a reasonable value for a large molecule. The ensemble resolution calculated using SASRES program, is 52 ± 4 Angstrom. (**B**) A representative SAXS data fitting for one GASBOR model. Red curve is the SAXS profile calculated from the resultant GASBOR structural model, while the black curve with error bar is the experimental SAXS data. The chi^2 between them is 0.17.

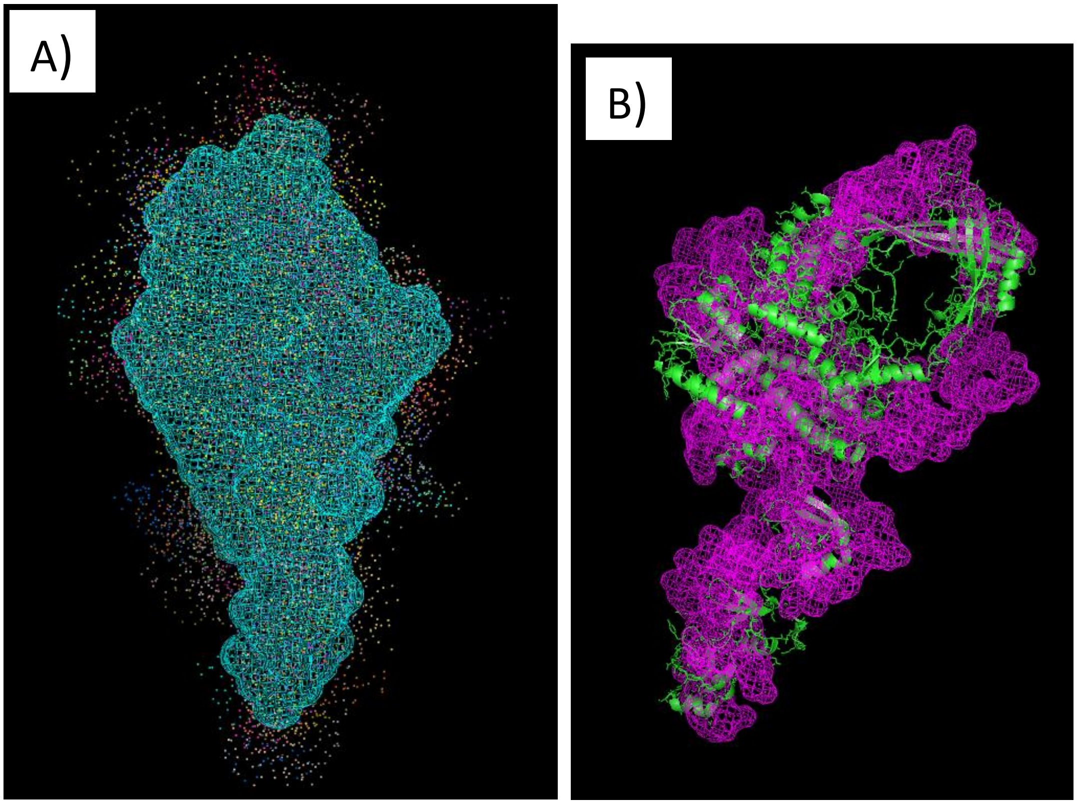

Figure S3. GASBOR models for MsmTop1-839t and superimposition to an atomic model. (**A**) Individual ab initio models (dots, each color represents one GASBOR model) calculated using program GASBOR and averaged model (cyan mesh) for MsmTop1-839. Program DAMAVER was used to average those individual models after removing "water molecules" in each individual GASBOR model. The normalized spatial discrepancy (NSD) of those models is: NSD = 2.0 ± 0.1. The ensemble resolution calculated using SASRES program, is 53 ± 4 Angstrom. (**B**) The superimposition of the most probable model (magenta mesh) in the GASBOR models described in **A**, and the structural model (green carton) with C-terminal domains pointing down. This structural model fits the SAXS model better.

Reference for program SASRES:

A.T. Tuukkanen, G.J. Kleywegt and D.I. Svergun (2016) Resolution of ab initio shapes determined from small-angle scattering. IUCrJ 3, 440-447.

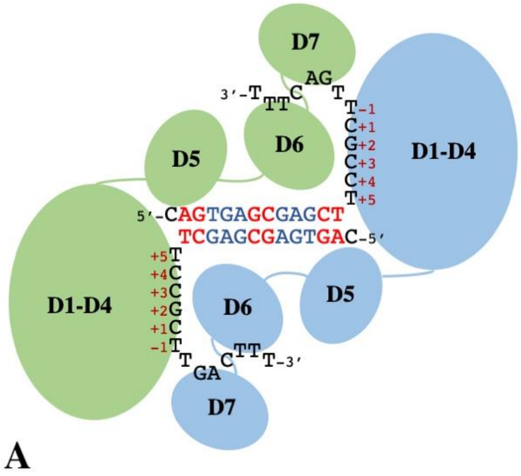

Figure S4. Schematic diagram of the DNA-protein complex in the crystal structure. (A) Schematic diagram of the formation of a dimer-like assembly of the MsmTOP1-839t/MTS2-25 complex in the crystal. The formation of the dsDNA part from oligo MTS2-25 is believed to be a consequence of high concentrations of both MsmTOP1-839t and MTS2-25 used under given crystallization condition. B) Schematic diagram of one MsmTOP1-839t monomer interacting with two ssDNA segments when the dsDNA region and the second MsmTOP1-839t monomer are removed from the dimer-like assembly.

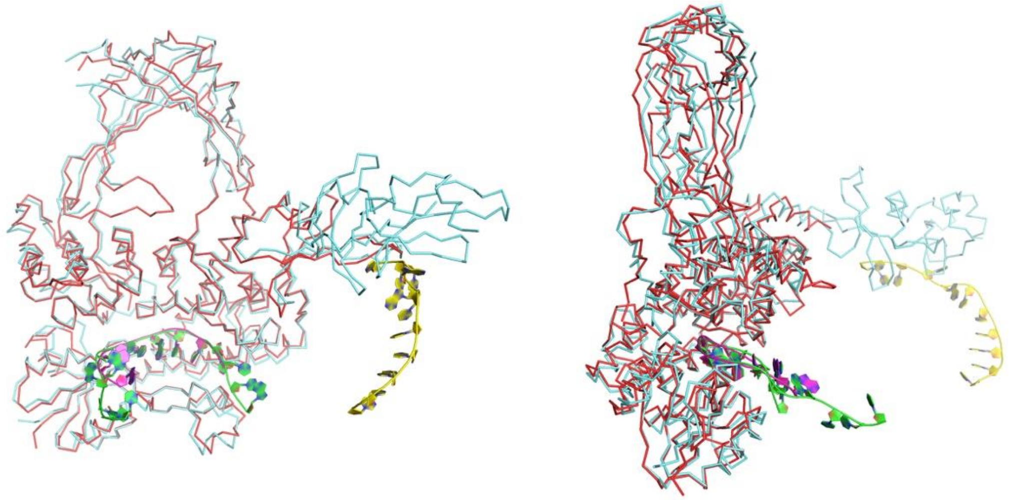

Figure S5. Front view (left) and side view (right) of the structural alignment (via Cα traces) of MsmTOP1-839t/MTS2-25 and MtbTOP1-704t/MTS2–11 (PDB code: 6CQI). All ssDNAs are drawn in cartoon format. MsmTOP1-839t is in cyan. The two MTS2-25 oligonucleotides in the MsmTOP1-839t/MTS2-25 complex are colored in magenta and yellow, respectively. MtbTOP1-704t is in red. Its bounded ssDNA MTS2–11 is drawn in green.

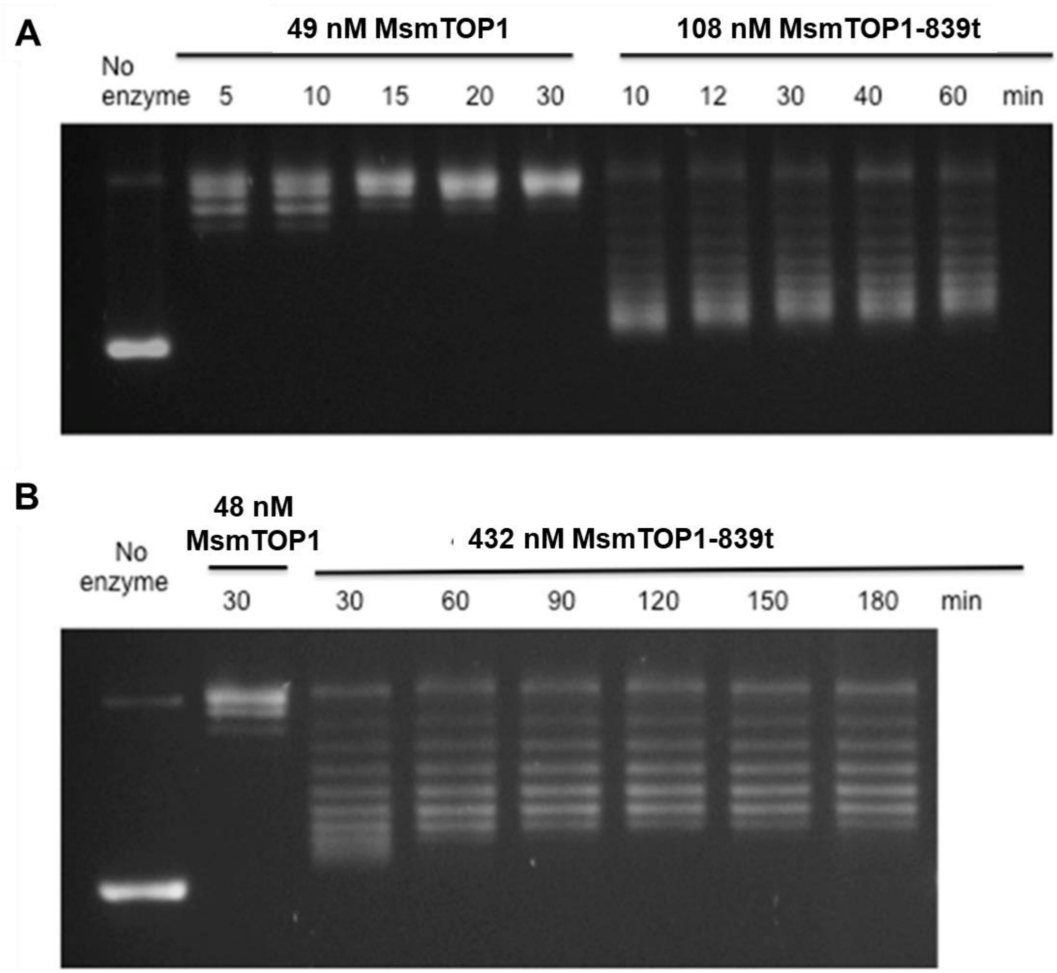

Figure S6. Time course of relaxation of negatively supercoiled plasmid DNA by MsmTOP1 and MsmTOP1-839t.

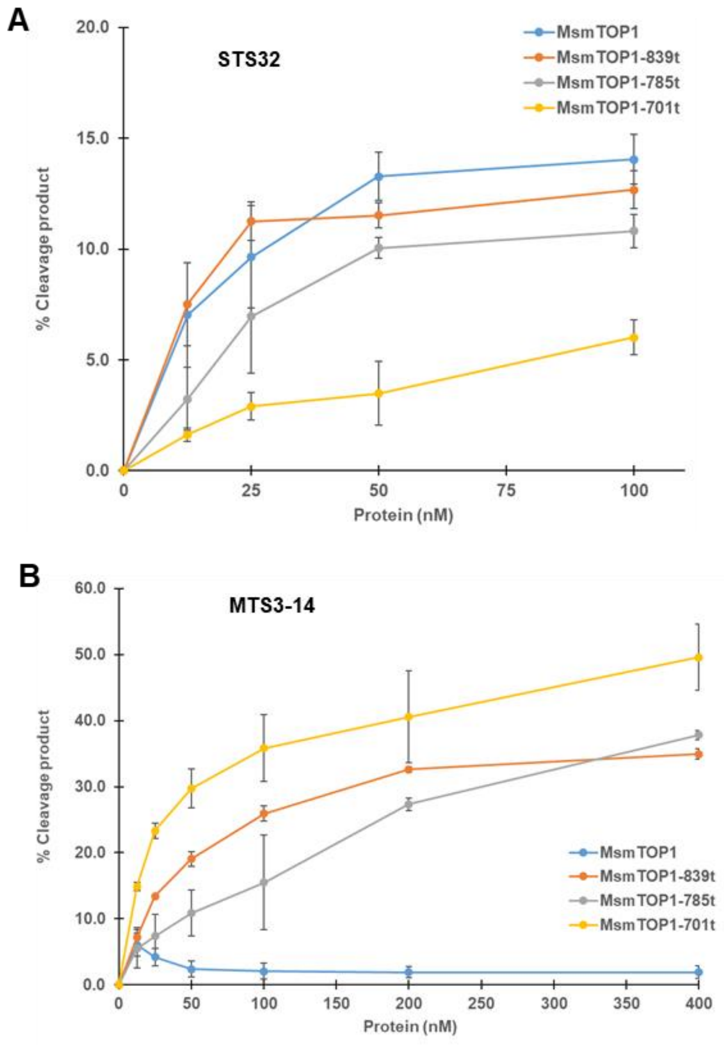

Figure S7. Cleavage of oligonucleotide substrate (A) STS32 (B) MTS3-14 by full length and truncated MsmTOP1. The percent of oligonucleotide cleaved by the enzymes was determined by densitometry analysis with the Alphaview software. The graphs represent the average and standard deviation from three independent experiments.

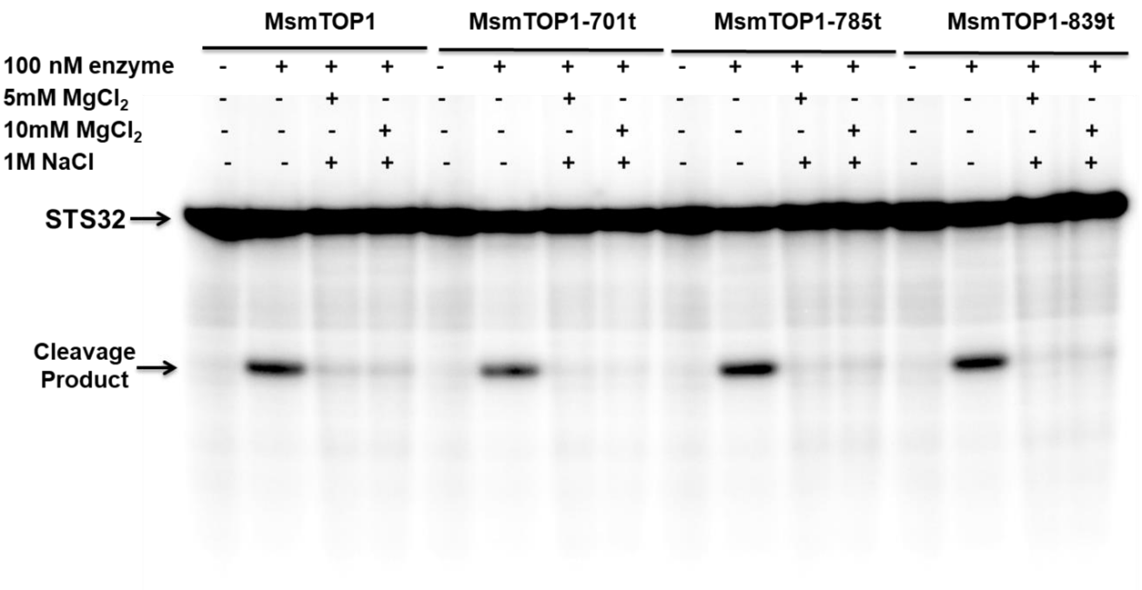

Figure S8. Religation assay of full length and mutant MsmTOP1 with C-terminal domain deletions. The 32-base oligonucleotide STS32 (100 nM) was first incubated with equal concentration of full length or truncated enzyme to generate the cleavage product before the addition of $MgCl_2$ plus NaCl to shift the cleavage-religation equilibrium towards DNA religation and dissociate the enzyme from the religated substrate.

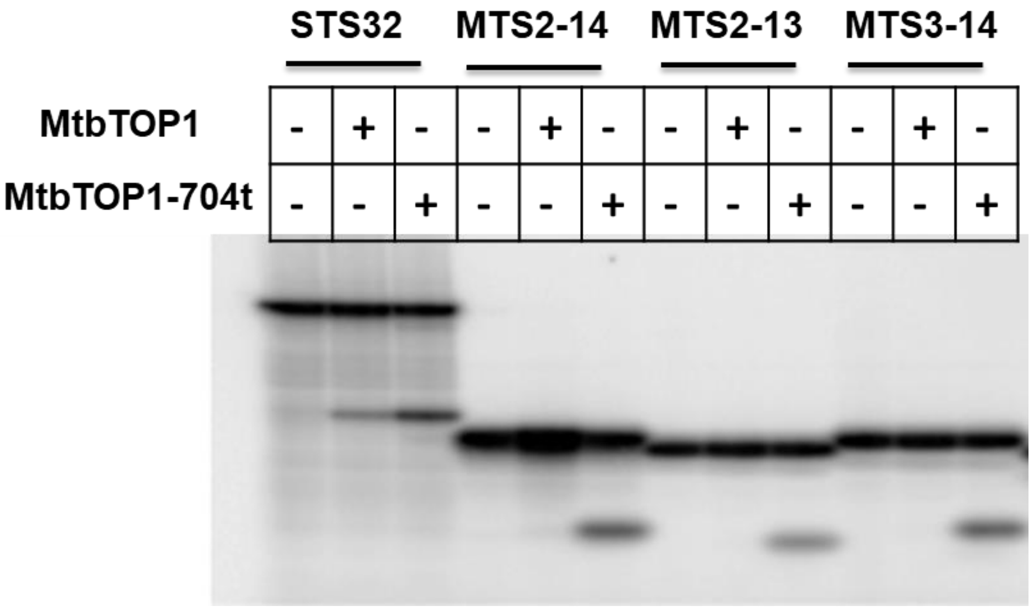

Figure S9. Cleavage activity assay of full length MtbTOP1 and MtbTOP1-704t at 2:1 protein:oligonucleotide ratio. MtbTOP1 and MtbTOP1-704t at 200 nM concentration were incubated with 100 nM of STS32, MTS2-14, MTS2-13, MTS3-14 at 37°C for 30 min.

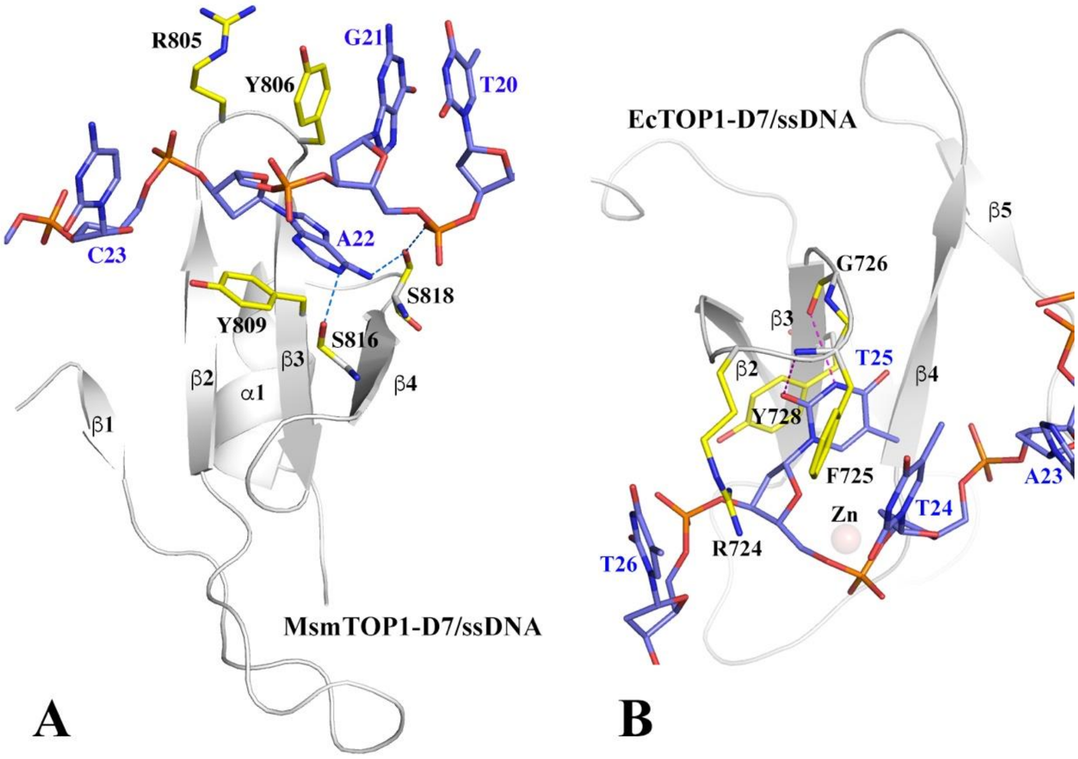

Figure S10. Comparison of ssDNA binding by C-terminal domain of MsmTOP1 and EcTOP1. (A) A ribbon diagram of MsmTOP1 D7 and its interaction with ssDNA as observed in MsmTOP1-839t/MTS2-25 structure. MsmTOP1 D7 represents a C-terminal domain of Topo_C_Rpt subgroup of topoisomerase I enzymes. (B) A ribbon diagram of EcTOP1 D7 and its interaction with ssDNA as observed in EcTOP1/O29-O29 structure (PDB 4RUL). EcTOP1 D7 represents a C-terminal domain of Topo_C_ZnRpt subgroup of topoisomerase I enzymes. Though Topo_C_Rpt and Topo_C_ZnRpt have different folds, each C-terminal domain commonly binds ssDNA on the open side of its β-sheet with ssDNA running across β-strands. As described in text, two conserved aromatic residues from each C-terminal domain form π-π stackings with the bases of ssDNA. Additional π-π stacking and/or other forms of interactions, including hydrogen bond, salt-bridge and π-cation interaction are involved in the interaction between an individual C-terminal domain and ssDNA.
